# Ultrasound education in the digital era: face-to-face vs. webinar-teaching of head and neck ultrasound theory—a prospective multi-center study

**DOI:** 10.3389/fmed.2025.1506260

**Published:** 2025-05-09

**Authors:** Johannes Matthias Weimer, Maximilian Rink, Marie Brandt, Luisa Symeou, Benjamin Philipp Ernst, Christoph Sproll, Alessandro Bozzato, Lukas Pillong, Johanna Helfrich, Andreas Weimer, Marie Stäuber, Holger Buggenhagen, Roman Kloeckner, Florian Recker, Thomas Beleites, Naglaa Mansour, Julian Künzel

**Affiliations:** ^1^Rudolf Frey Learning Clinic, University Medical Center of the Johannes Gutenberg University Mainz, Mainz, Germany; ^2^Department of Medicine, University Medical Center of the Johannes Gutenberg University Mainz, Mainz, Germany; ^3^Department of Otorhinolaryngology, Head and Neck Surgery, University Hospital Regensburg, Regensburg, Germany; ^4^Department of Otorhinolaryngology, University Medical Center Frankfurt, Frankfurt, Germany; ^5^Department of Oral and Maxillofacial Surgery, University Hospital Düsseldorf, Heinrich-Heine-University Düsseldorf, Düsseldorf, Germany; ^6^Department of Otorhinolaryngology, University of Saarland, Homburg, Germany; ^7^Center of Orthopedics, Trauma Surgery, and Spinal Cord Injury, Heidelberg University Hospital Heidelberg, Heidelberg, Germany; ^8^Institute of Interventional Radiology, University Hospital Schleswig-Holstein—Campus Lübeck, Lübeck, Germany; ^9^Department of Obstetrics and Prenatal Medicine, University Hospital Bonn, Bonn, Germany; ^10^Department of Otorhinolaryngology, Head and Neck Surgery, University Hospital Carl Gustav Carus Dresden, Dresden, Germany; ^11^Department of Otorhinolaryngology, Head and Neck Surgery, Medical Center-University of Freiburg, Freiburg, Germany

**Keywords:** head and neck ultrasound, certified ultrasound education, digitalization, digital transformation, blended learning, webinar-teaching, face-to-face-teaching

## Abstract

**Introduction:**

Digitalization offers significant potential benefits to ultrasound education. This study compares the effectiveness of webinar teaching against face-to-face teaching in providing theoretical competencies in certified head and neck ultrasound (HNUS) courses.

**Patients and methods:**

This prospective, controlled, multicenter study was conducted in 2023 at three universities with certified HNUS courses. One course used webinar lessons (S), and the others used face-to-face teaching (C). The control group courses (C) were held on two consecutive days. The first day of the study group course was held as a webinar (S) 1 week before the second day and was also recorded for preparatory purposes. All participants completed three assessments: a pre-course self-evaluation (Evaluation^pre^), a post-course self-evaluation (Evaluation^post^), and a post-course theory test (Theory Test^post^). The evaluations used a Likert scale (1–7) to record the participants’ subjective assessments of competencies and attitudes toward webinar teaching. Theory Test^post^ included multiple-choice and free-answer questions on the sonographic pathologies of lymph nodes, the soft tissue of the neck, and salivary glands. A group of inexperienced medical students (V) completed the Theory Test^post^ for validation purposes.

**Result:**

128 data sets were analyzed (31 S; 30 C; 47 V). Both groups, S and C, rated their competencies after the courses significantly higher than before (*p* < 0.01) but at a similar level in comparison with each other (*p* = 0.34). Both groups supported teaching theoretical content through webinars (S: 6.7 ± 0.5 vs. C: 6.2 ± 0.9). Both groups achieved similar results in the Theory Test^post^ (*p* = 0.54), significantly outperforming the validation group (*p* < 0.001).

**Conclusion:**

Our data suggest that webinars can be an effective alternative to face-to-face lessons in teaching theoretical competencies in HNUS. Participants gave overall positive evaluations of digital teaching methods. Our findings support evidence that digital learning methods are valuable for modern ultrasound education.

## Introduction

Ultrasound is used by physicians across a variety of specialties to provide rapid access to diagnostic information. It is cost-effective, ubiquitous, and does not expose patients to radiation. It can immediately confirm or exclude a suspected diagnosis and thus direct further diagnostic pathways. It can also guide interventional procedures, making them safer, more accurate, and easier to perform quickly and at scale (1–4). Technological advancements have made ultrasound units more compact, easily maneuverable, and less expensive, and they now provide high-resolution images. Ultrasound devices can now fit in a coat pocket, making them particularly useful in emergency medicine (5, 6).

Head and neck ultrasound (HNUS) is routinely performed by otorhinolaryngologists, head and neck surgeons, maxillofacial surgeons, radiologists, and internal physicians. Ultrasound education is therefore crucial in these disciplines and forms part of their residency programs. Nevertheless, not every department has specialists to teach their residents adequately. To educate physicians in HNUS, the German Society of Ultrasound in Medicine (DEGUM) additionally certifies specialists in HNUS and offers certified basic and advanced courses in HNUS to teach theoretical and practical ultrasound skills (7).

During the COVID-19 pandemic, ultrasound education adopted digital education methods, including webinars, to ensure conformity with social distancing measures (8, 9). Among them, webinars have shown particular promise due to their flexibility, reduced travel burden, and potential to standardize content delivery (8, 9). Additionally, institutions such as the National Association of Statutory Health Insurance for Physicians called for “more flexibility to participate in ultrasound courses.” They scheduled a three-year test phase to evaluate the comparative effectiveness of digitally-supported ultrasound courses (10).

Educational research has thus recently studied a range of innovative digital teaching methods in ultrasound education (8, 9, 11–13). These innovations include tele-guided ultrasound training, where an expert remotely guides learners during live scanning sessions (11); video-based instructions, often used for practical demonstrations (9); and structured webinars or online E-learning modules, which allow flexible access to theoretical content (8, 12, 13). As workforce shortages and time constraints limit access to traditional continuing medical education, these digital approaches may offer practical solutions for postgraduate training environments.

In undergraduate ENT education, online lectures were equally effective in conveying emergency knowledge (14). E-learning programs implemented during the COVID-19 pandemic maintained high learner engagement and perceived competence levels (15).

In postgraduate medical training, virtual OSCE preparation led to performance outcomes similar to conventional face-to-face formats, supporting structured digital alternatives (16). Likewise, web-based modules in pediatric lung ultrasound enabled novice learners to achieve theoretical knowledge levels equivalent to those taught in classroom settings (17).

In the field of HNUS, video-based and telepresence-guided course formats have also been developed, showing positive learner feedback and perceived improvements in competence (18, 19). However, these studies primarily assessed subjective outcomes, such as self-confidence and course satisfaction, rather than objective performance and are focused on undergraduate education. Further work on digitally supported ENT training demonstrated successful implementation for teaching practical skills while highlighting the continued importance of direct hands-on validation for skill acquisition (20). Some studies have explored webinar-based teaching in general ultrasound education (8, 13). One such study implemented a pandemic-adapted blended learning model that combined webinars with small-group bedside teaching in a small-group format (8). Although this approach was well received and demonstrated the feasibility of webinar integration, the evaluation relied primarily on subjective learner feedback, and no objective testing was used to assess knowledge acquisition.

Despite the growing body of research on digital education strategies, robust and high-quality evidence on the effectiveness of webinar-based teaching in HNUS remains scarce. Importantly, broader educational reviews (21) confirm that webinars can effectively foster knowledge acquisition and behavioral change—but also emphasize that outcomes vary by discipline, learner level, and instructional design, underscoring the need for domain-specific evidence. While initial experiences with fully virtual DEGUM-certified HNUS courses have shown promising levels of learner acceptance and feasibility (12), systematic evaluations using controlled designs and comparative data to in-person formats are still lacking.

The present study aims to address this gap by providing robust comparative evidence on the effectiveness and acceptance of webinar-based compared to traditional face-to-face theoretical instruction within certified head and neck ultrasound courses. We hypothesize that the participants will value the digital aspects of the HNUS training and evaluate them positively. By assessing learning outcomes with a structured theory test and ascertaining the participants’ attitudes toward digital learning methods through a pre-and post-course evaluation, this study will evaluate the effectiveness and desirability of digitized HNUS courses.

## Materials and methods

### Study design and recruitment of participants

**Figure 1:**
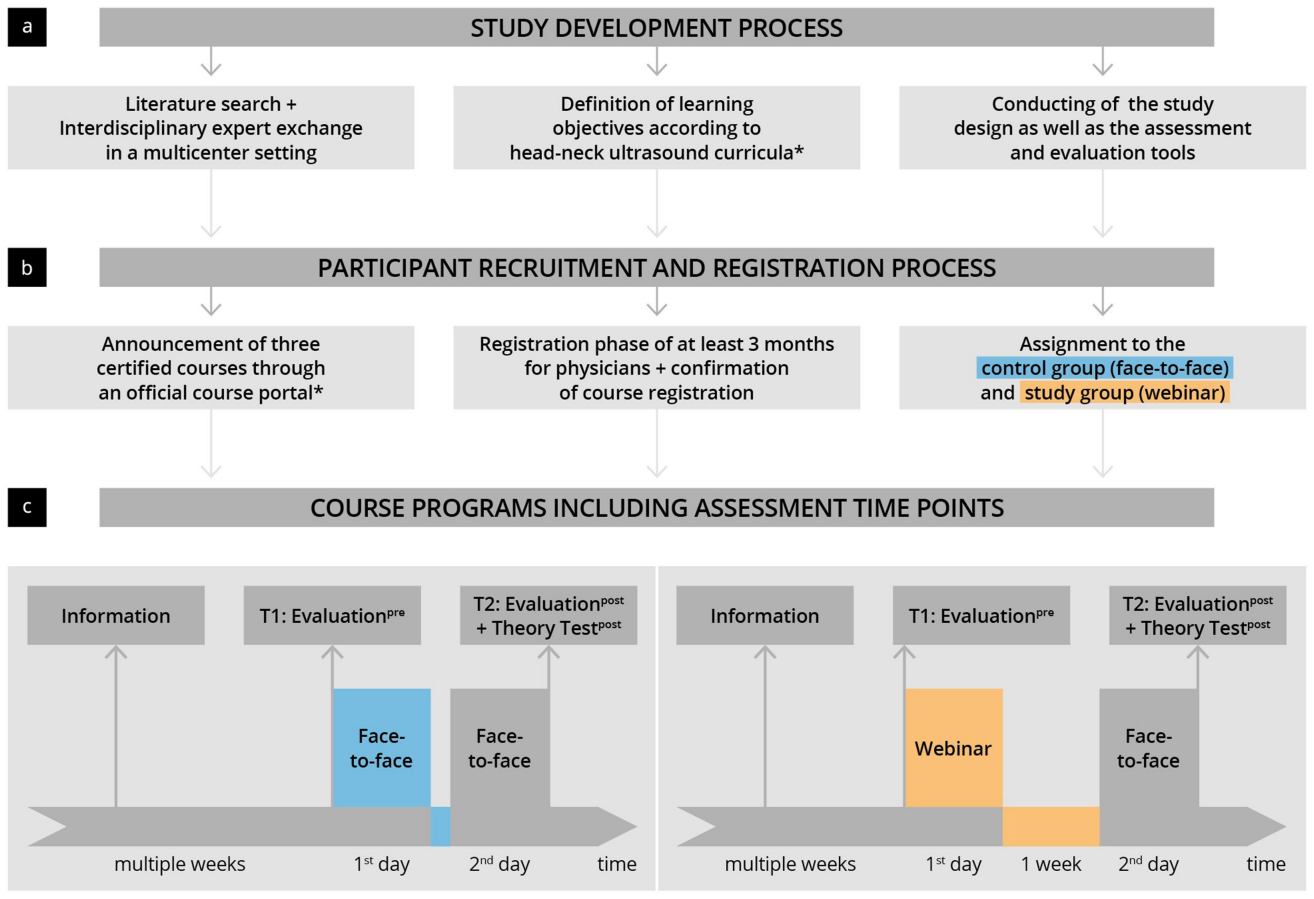
Illustration of the study development and implementation process. **(a)** An interdisciplinary planning and development phase was followed by panel **(b)** participant recruitment and panel **(c)** implementation of the prospective controlled study as part of certified HNUS courses. ^*^The German Society for Ultrasound in Medicine (DEGUM).

This prospective, controlled, multicenter study was planned and implemented at three university sites in 2023 (see Figure 1) (22, 23).

The intention was to compare the effectiveness of teaching theoretical competencies through webinars and face-to-face teaching within DEGUM-certified HNUS training courses. To this end, three ultrasound courses from three providers were selected through tendering via DEGUM’s official platform (“DEGUM’s course portal”). One of the three courses taught theoretical content through webinars instead of face-to-face learning. Participants in this webinar group became the study group (S), and the other two groups became controls (C). Participants were made aware of the different teaching modalities in the course descriptions. Participants were invited to participate in the study at the beginning of the course. Participation involved completing an evaluation before the start of the course (Evaluation^pre^ at time T1) and an evaluation at the end of the course, as well as a written assessment concerning ultrasonographic pathologies in the head and neck region (Evaluation^post^ and Theory Test^post^ at time T2). The study was approved by the Regensburg Ethics Committee and received a waiver (date: 20/12/2022).

The inclusion criteria were consent to participate, full participation in the course, and the completion of the evaluation forms and assessment. The study’s primary endpoints were defined as theoretical competence, measured by a written exam at the end of the course, and a subjective increase in competencies, measured through self-evaluations before and after the course. Secondary endpoints relate to the attitude, motivation, and acceptance of digitized HNUS training concepts through self-evaluations before and after the course.

### Course conceptions

The courses were designed and conducted according to the guidelines of the head–neck section of DEGUM and were certified as an “advanced course” (7). The learning objectives of the course were based on the recommended curriculum (7). Before the start of the three courses, the instructors ensured that the contents of the respective courses aligned. The control group course consisted of two consecutive days of face-to-face lessons, alternating between theoretical lectures in the plenary and practical exercises. The study group’s course included a full day of webinar lessons via video conference software and a separate day of face-to-face lessons for the practical exercises a week later. During the webinar, attendance was checked by recording the names of online participants before each lecture. Participants could ask questions at any time through the microphone or chat functions. In addition, active participation was encouraged, with open discussions after the respective lectures. All the webinar lectures were recorded and made available to the participants after the first day of the course in preparation for the face-to-face practice day. All courses were supervised by a certified course instructor (DEGUM Level III).

### Assessment tools

The evaluations and test tools were created by a panel of ultrasound and teaching experts, relying on current recommendations and literature (24–31).

In the self-evaluations (Evaluation^pre^ and Evaluation^post^), questions were clustered according to category: “personal data”; “previous experience”; “course preparation”; “motivation/expectations”; “subjective competencies assessment”; “user behavior and attitudes to digital media and teaching concepts, including webinar teaching”; and “overall course evaluation.” These questions were answered either on a seven-stage Likert scale (1 = does not apply at all; 7 = applies entirely), dichotomously (“yes”/“no”), or in free-text fields (30, 31). The written Theory Test^post^ (max. 54 points) assessed the participants’ progress in recognizing “lymph node pathologies” (max. 12 points); “cervical soft tissue pathologies” (max. 26 points); and “salivary gland pathologies” (max. 16 points). These areas match the defined learning objectives of DEGUM’s HNUS development-course curriculum (7). The questions (see Supplementary material 1) required mostly single or multiple-choice answers with some “very short answer” questions (24–29). The test took approximately 40 min to complete. The answer key was defined by consensus of 6 DEGUM III-certified HNUS experts, each having more than 10 years of experience in the field. A control group of inexperienced students also completed the Theory Test^post^ to validate it (32). The participants were recruited as part of the university’s student ultrasound program.

### Statistical methods

Data of the self-evaluations and theoretical learning success checks were manually evaluated using Microsoft Excel before analysis in R studio (33) with R 4.0.3 (34). Binary and categorical baseline parameters are expressed as absolute numbers and percentages. Continuous data are expressed as median and interquartile range (IQR) or mean and standard deviation (SD). Categorical parameters were compared using the chi-squared test, and continuous parameters using the Mann–Whitney test. *p*-values < 0.05 were considered statistically significant. For this study, a power analysis was performed to determine the sample size required for statistical significance. Based on an expected effect size of 0.8, a significance level of 0.05, and a desired power of 0.90, the calculated sample size was set at 68 participants (34 in each group).

## Results

### Participants

91 participants across three centers were invited to participate (see Supplementary material 2). Ultimately, 61 participants met the inclusion criteria (*n* = 30 in the control group and *n* = 31 in the study group). In addition, the Theory Test^post^ results of a group of inexperienced students (*n* = 47) were used for validation. Table 1 shows the detailed baseline characteristics of the participants. Women were the majority of participants in the study group, unlike in the control group (*p* = 0.02). Otherwise, the demographic characteristics and pre-education profiles were roughly equivalent. Both groups had a similar average age (control 32.2 ± 4.9 vs. study 33.1 ± 5.3; *p* = 0.72), were mainly residents (control: 73% vs. study 84%; *p* = 0.89), had already completed an introductory course in HNUS (Control: 70% Vs. study 94%; *p* = 0.48), and reported completing a similar amount of previous ultrasound examinations (control: 205 ± 197 vs. study 282 ± 391; *p* = 0.46). A majority in both groups had used digital teaching media before (control: 73% vs. study 61%; *p* = 0.05) and had not previously attended any ultrasound-specific webinars (control: 77% vs. study 97%; *p* = 1.0).

**Table 1:** Baseline characteristics and previous experience of participants in the study-and control-groups.

Item	Control-group *N* = 30	Study-group *N* = 31	*p*-value
Gender			0.02
Female (*n*;%)	10 (33%)	19 (61%)	
Male (*n*;%)	15 (50%)	12 (39%)	
n.a. (*n*;%)	5 (17%)	0	
Age (mean ± SD) in years	32.2 ± 4.9	33.1 ± 5.3	0.72
Level of training			0.89
Resident (*n*;%)	22 (73%)	26 (84%)	
Board-certified specialist (*n*;%)	2 (7%)	3 (10%)	
Senior physician (*n*;%)	1 (3%)	2 (6%)	
n.a. (*n*;%)	5 (17%)	0	
Specialty			0.88
Ear, nose, and throat medicine (*n*;%)	18 (60%)	24 (77%)	
Oral and maxillofacial surgery (*n*;%)	7 (23%)	7 (23%)	
n.a. (*n*;%)	5 (17%)	0	
Year of residency (mean ± SD)	3.3 ± 1.9	4.7 ± 3.8	0.10
Ultrasound courses attended			0.48
Yes (*n*;%)	21 (70%)	29 (94%)	
No (*n*;%)	4 (13%)	2 (6%)	
n.a. (*n*;%)	5 (17%)	0	
How long ago completed a basic ultrasound course in HNUS (mean ± SD) years	1.7 ± 1.5	2.6 ± 1.8	0.10
Number of ultrasound examinations (mean ± SD)	204.75 ± 197.49	281.84 ± 391.05	0.46
Participation in webinars in general			0.42
Yes (*n*;%)	16 (53%)	24 (77%)	
No (*n*;%)	9 (30%)	7 (23%)	
n.a. (*n*;%)	5 (17%)	0	
Time extent of previous webinar participation (mean ± SD)	31.33 ± 29.35	24.75 ± 21.5	0.49
Participation in ultrasound webinars			1.0
Yes (*n*;%)	1 (3%)	1 (3%)	
No (*n*;%)	23 (77%)	30 (97%)	
n.a. (*n*;%)	6 (20%)	0	
Preparation time (mean ± SD) in h	1.9 ± 3.4	2.6 ± 3.0	0.42
Use of digital media			0.05
Yes (*n*;%)	22 (73%)	19 (61%)	
No (*n*;%)	3 (10%)	12 (39%)	
n.a. (*n*;%)	5 (17%)	0	
Use of digital ultrasound media			0.33
Yes (*n*;%)	8 (26%)	15 (48%)	
No (*n*;%)	17 (57%)	16 (52%)	
n.a. (*n*;%)	5 (17%)	0	

### Subjective competencies assessment (T1 + T2)

**Figure 2:**
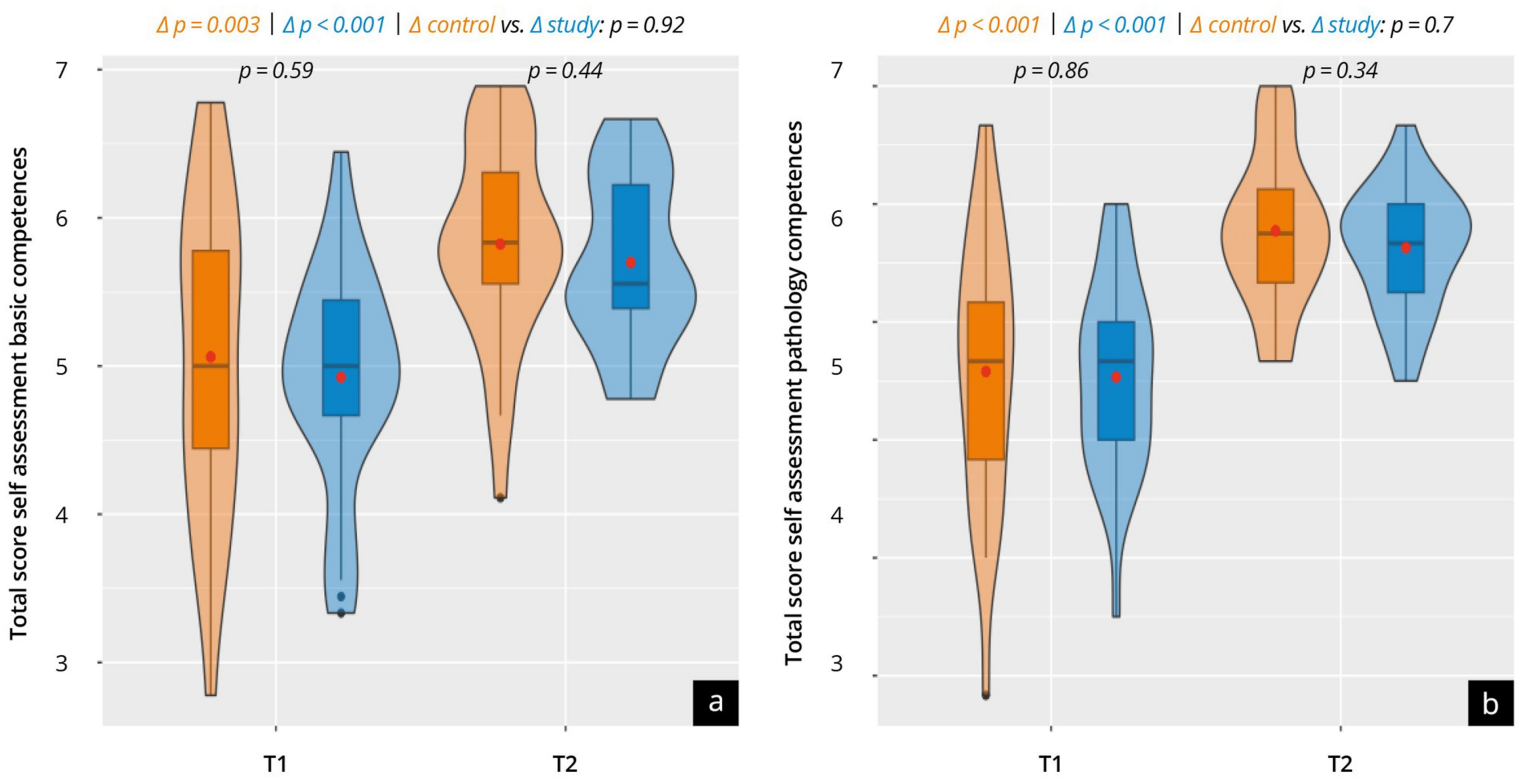
Results of the subjective assessment of competencies in a group comparison between the control group (orange) and the study group (blue) at time points T1 and T2. The violin plots present the total score for **(a)** the basic competencies and **(b)** the pathology competencies. The results of the sub-categories are listed in Supplementary material 3.

The results of the subjective competencies assessment before (Evaluation^Pre^, T1) and after the course (Evaluation^Post^, T2) are shown in Figure 2 and Supplementary material 3. Before the start of the course, both groups similarly rated their basic skills (control 5.1 ± 1.1 vs. study 4.9 ± 0.8; *p* = 0.6) and their ability to recognize pathological findings similarly (control 4.6 ± 1.1 vs. study 4.5 ± 0.8; *p* = 0.86). After completion of the course (T2), both groups rated their competencies significantly higher (*p* < 0.01) and achieved an equivalent level concerning the basic (control 5.8 ± 0.7 vs. study 5.7 ± 0,57; *p* = 0.4) and pathology competencies (control 5.8 ± 0.6 vs. study 5.6 ± 0.5; *p* = 0.34). This trend was also observed in the sub-categories surveyed, with the most significant skill growth in the categories “image optimization” (*Δ* control 1.7 ± 1.5 vs. Δ study 1.1 ± 1.3; *p* = 0.09), “pathology of the salivary glands” (Δ control 1.2 ± 1.2 vs. Δ study 1.0 ± 1.0; *p* = 0.49), “paranasal sinuses” (Δ control 1.6 ± 1.7 vs. Δ study 1.3 ± 1.8; p = x).

### Attitude toward digitalization and digital teaching methods (T1 + T2)

**Figure 3:**
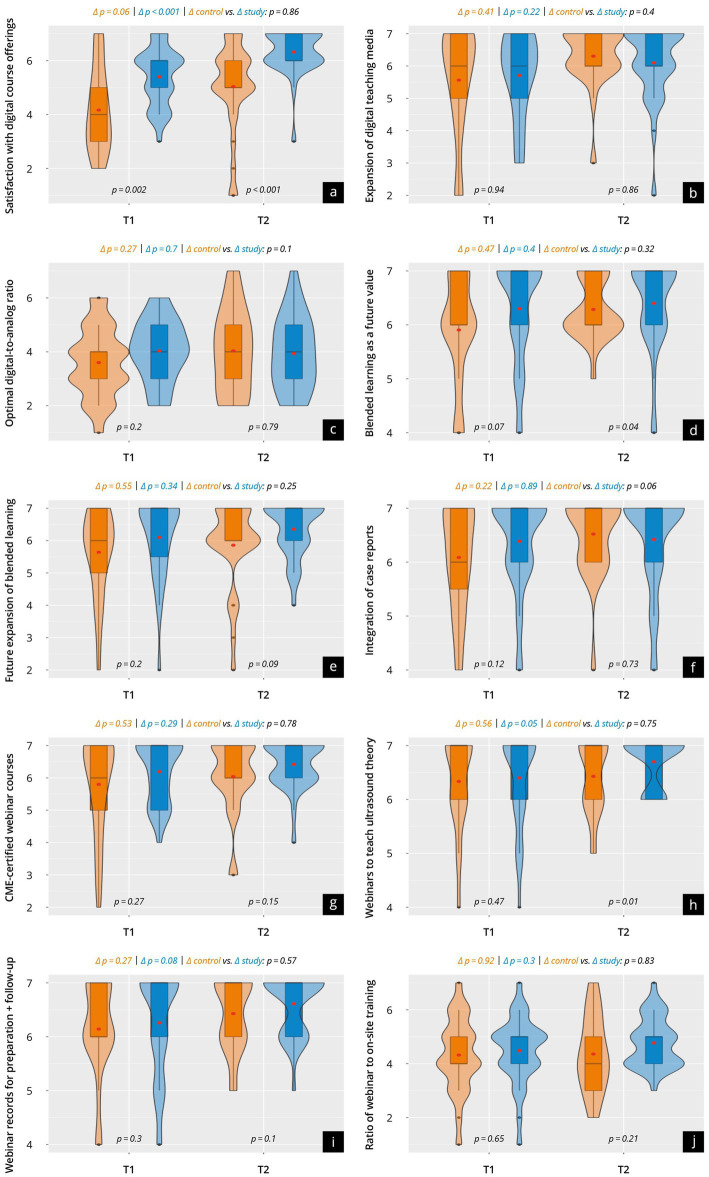
Results of the evaluation of attitudes toward digital training concepts and prospects in a group comparison at time points T1 and T2. The violin plots present the results of the following surveyed items: **(a)** Satisfaction with digital course offerings; **(b)** Expansion of digital teaching media; **(c)** Optimal digital-to-analog ratio; **(d)** Blended learning as a future value; **(e)** Future expansion of blended learning; **(f)** Integration of case reports; **(g)** CME-certified webinar courses; **(h)** Webinars to teach ultrasound theory; **(i)** Webinar records for preparation and follow-up; **(j)** Ratio of webinar to on-site training.

Data on the participants’ attitudes toward digitalization and digital teaching methods are presented in Figure 3 and Supplementary material 3. Both groups evaluated the surveyed items in high-scale ranges in T1, with a tendency toward higher values in the study group. There were strong approval ratings for “extending blended learning” (control 5.6 ± 1.4 vs. study 6.2 ± 1.2; *p* = 0.07), “transmission of theoretical content through webinar teaching” (control 6.0 ± 1.44 vs. study 6.3 ± 1.2; *p* = 0.47) and “webinar recording for pre-and post-course preparation” (control 5.9 ± 1.4 vs. study 6.3 ± 0.97; *p* = 0.3). Slightly lower with statistical significance was the “satisfaction with the current digital teaching offer in ultrasound education” evaluated by both groups (control 4.2 ± 1.6 vs. study 5.4 ± 1.1; *p* = 0.002). The study asked participants to give their preferred mix of teaching methods on a 7-point Likert scale, where courses taught solely through webinars were designated = 7 and those taught solely in person were designated = 1. Both groups tended to slightly prefer webinar-led learning (control 4.3 ± 1.4 vs. study 4.5 ± 1.2; *p* = 0.65).

After the course (T2), both the control group and the study group evaluated the respective items in slightly higher scale ranges, with higher values tending to be recorded in the study group. Particularly high approval was recorded concerning “increased offer of webinar training with CME certifications” (control 6.0 ± 1.1 vs. study 6.4 ± 0.7; *p* = 0.15) and “webinar recording for course pre-and post-preparation” (control 6.3 ± 0.8 vs. study 6.6 ± 0.6; p = 0.1). “Future blended learning” (control 5.8 ± 1.4 vs. study 6.4 ± 0.8; *p* = 0.04) and “transmission of theoretical content through webinar teaching” (control 6.2 ± 0.9 vs. study 6.7 ± 0.5 *p* = 0.01) were rated very positively in both groups, but significantly higher in the study group.

### Course concept evaluation (T2)

**Figure 4:**
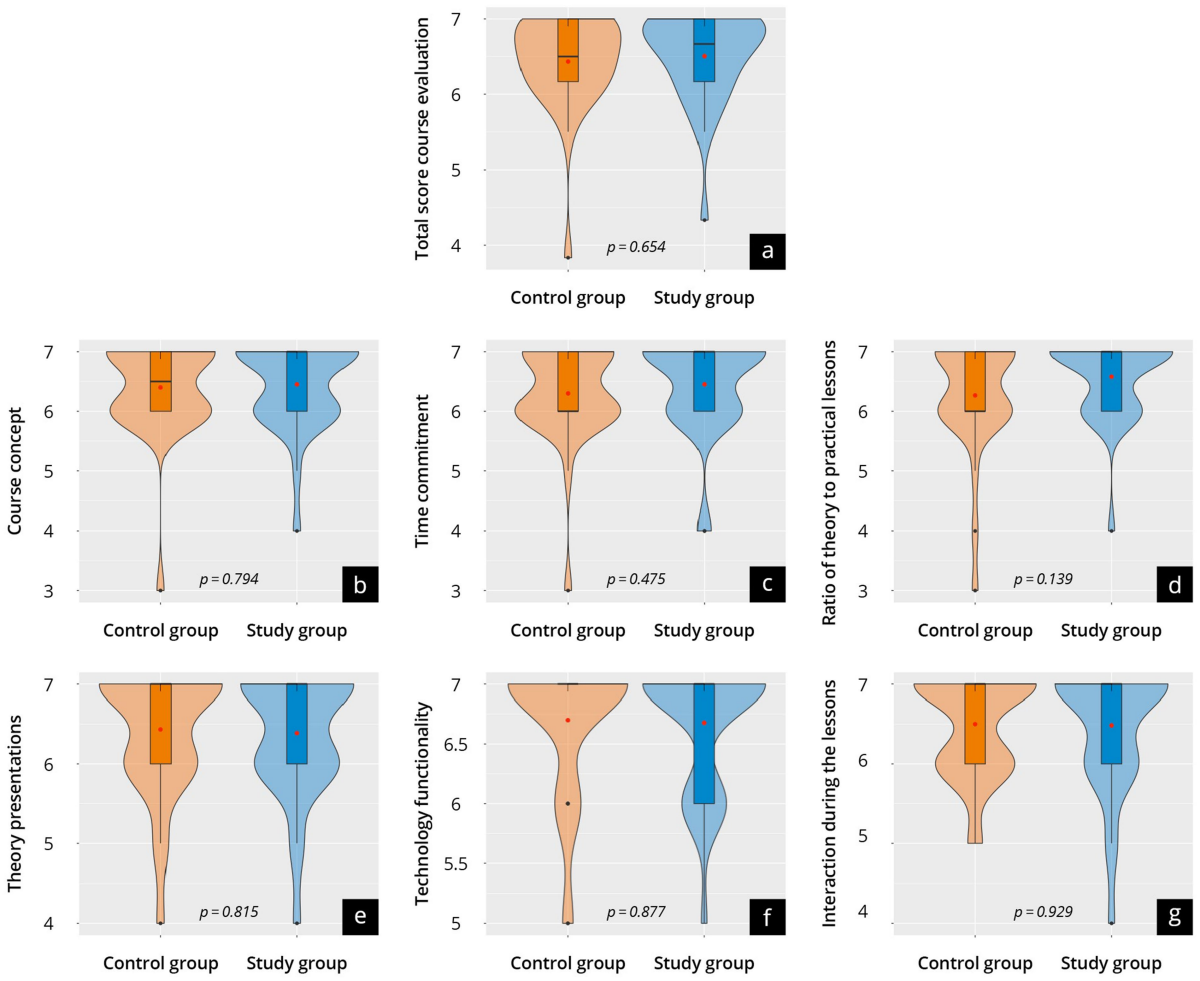
Results of the course evaluation in a group comparison between the control group (orange) and the study group (blue) at time point T2. The violin plots present **(a)** the total score, as well as **(b)** the course concept; **(c)** the time commitment; **(d)** the theory-practice ratio; **(e)** live lectures; **(f)** the functionality of technology; **(g)** interaction in class.

The results of the overall assessment of the courses and the webinar itself are represented in Figure 4 and Supplementary material 4. Both groups evaluated their overall course very positively, with no significant difference (control 6.4 ± 0.7 vs. study 6.5 ± 0.6; *p* = 0.65) across the groups. The study group also rated the webinar very positively (6.3 ± 0.7).

### Results of the Theory Test^post^ (T2)

**Figure 5:**
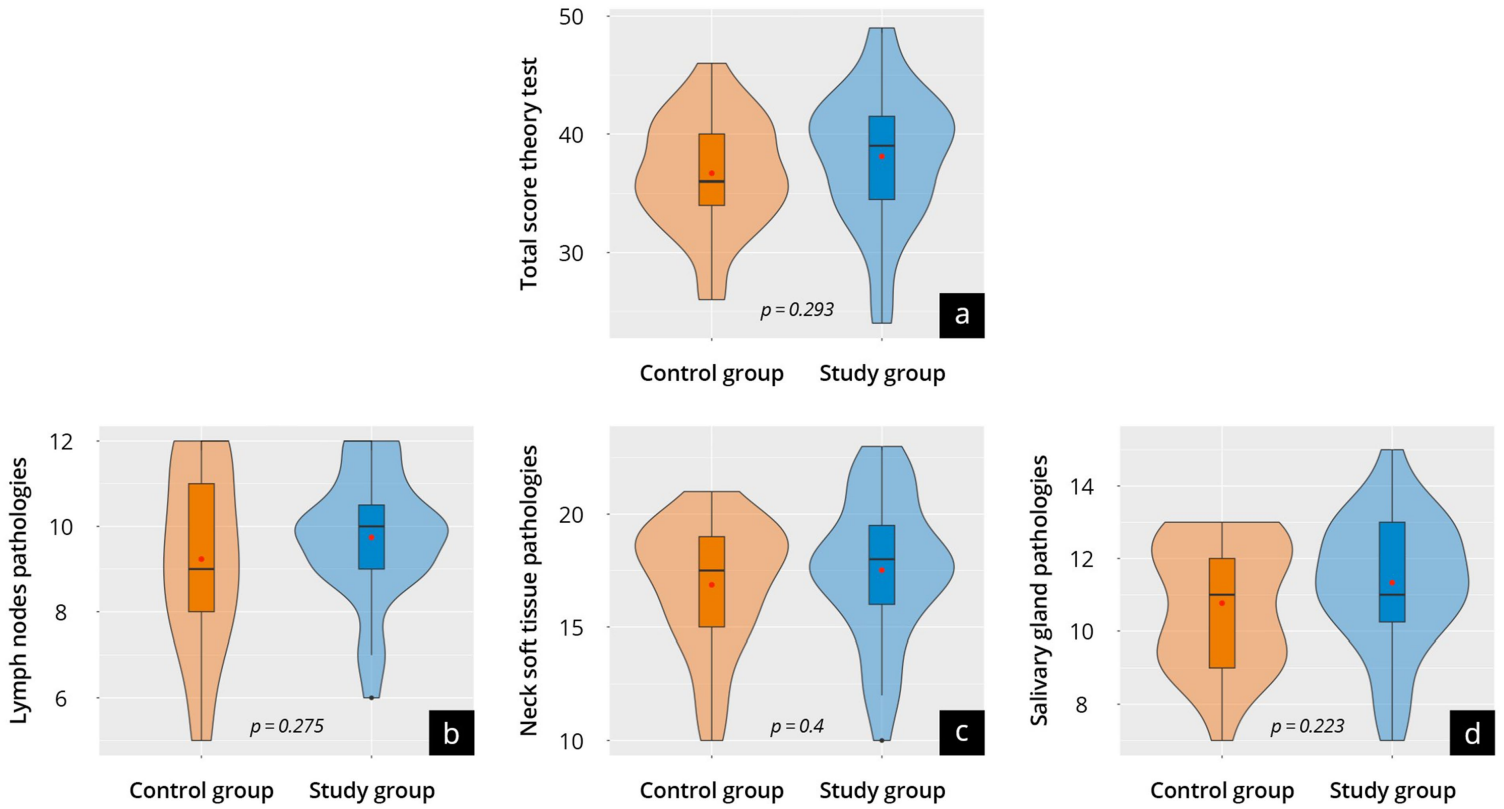
Results of the Theory-Test^post^ of the control group (orange) and study group (blue) at time point T2. The violin plots present **(a)** the total score and the scores in the relevant areas of expertise, namely: **(b)** lymph node pathologies; **(c)** soft tissue pathologies of the neck; and **(d)** salivary gland pathologies. The results of the individual questions are listed in Supplementary material 6.

The results of the post-course theoretical exam (Theory Test^post^) are presented in Figure 5 and Supplementary material 5. Overall, both groups achieved comparable test results (control 36.7 ± 4.7 vs. study 38.1 ± 5.7; *p* = 0.54). Both groups performed similarly in the “pathologies of the lymph nodes” (*p* = 0.28), “pathologies of the soft tissues of the neck” (*p* = 0.4), and “pathologies of salivary glands” (*p* = 0.22) subcategories.

### Test validation

The Theory Test^post^ results for the validation group, including the demographic profile of the participants, are shown in Supplementary material 6. Overall, the validation group scored significantly lower (*p* < 0.001) than the control and study groups.

## Discussion

This multicenter, prospective study provides the first structured and comparative investigation of webinar-based versus traditional face-to-face theoretical instruction within certified head and neck ultrasound education. Conducted across multiple centers and embedded in accredited courses, the study offers a real-world evaluation under standardized conditions. By simultaneously assessing subjective learning experience, objectively measured theoretical level of knowledge, and learner attitudes, it delivers a multidimensional perspective on the effectiveness and acceptance of digital teaching formats in a highly specialized, postgraduate training context.

Our results demonstrate that webinar-based teaching is not only equivalent to face-to-face instruction in promoting theoretical knowledge and self-reported competency development but is also highly accepted among learners. Rather than aiming to replace face-to-face learning, this study highlights the potential of webinars to expand and enhance existing educational models—particularly as part of blended learning concepts. This is particularly relevant in light of increasing demands for flexible and scalable educational formats. The use of a structured theory test ensures that outcome measurements go beyond perception and are anchored in objective performance data. This study addresses a critical shortcoming of earlier research, which often focused on undergraduates or not head–neck-specialized content areas, relied solely on subjective outcomes, or lacked controlled comparisons (8, 11–13).

Most importantly, this study contributes urgently needed evidence for the purposeful integration of digital teaching formats into certified continuing education ultrasound programs. This is particularly relevant for regulatory frameworks such as those currently piloted by certifying institutions. Our results provide a quality-assured, data-driven foundation for such initiatives and support the development of future-ready certification structures that align with ongoing digital transformation in medical education. In the following sections, we discuss the implications of these findings concerning the future integration of blended learning concepts into certified ultrasound education, the relative effectiveness and acceptance of webinar-based versus face-to-face theoretical instruction, and the role and limitations of digital formats in teaching practical sonographic skills.

### “Blended learning”

Blended learning integrates digital teaching and online media with in-person instruction and was developed to successfully minimize the educational disruption of the COVID-19 pandemic (35–45). Although blended learning has been gradually incorporated into undergraduate ultrasound education worldwide, this has not been the case in postgraduate medical education (9, 10, 46–49). Meanwhile, few studies have compared traditional sonography training models with digital training (13, 18, 19, 35, 49).

A blended-learning training model has already proven successful in musculoskeletal sonography and point-of-care ultrasound and was evaluated well compared to conventional, in-person training (13, 35). Comparably, HNUS education has increasingly implemented digital strategies, especially after the COVID-19 pandemic. Results of preliminary studies suggest digital instruction in HNUS effectively builds competencies. Learners receive it positively, particularly when deployed as part of a blended learning approach (12, 18–20, 42). Nevertheless, digital learning is far from universal in HNUS teaching.

Our study data supports findings that HNUS webinar teaching is well received and comparably effective to traditional in-person teaching in imparting theoretical competencies. Unlike other studies, our study explicitly measures for and depicts theoretical competence increases through a differentiated theoretical test with structured multiple-choice and free-text questions. Additionally, the use of a control group and a test validation group strengthens our results in comparison to previous studies. The positive self-evaluation and course evaluation results of both course groups (i.e., control and study) and the positive attitude toward digitalization suggest a need for digitalization in head and neck sonography education, especially in the integration of webinars.

While the time gap between the webinar and the in-person practical session may have affected performance by enabling extended preparation, this reflects a didactic advantage of blended learning. It offers learners flexible opportunities to revisit theoretical content. It reinforces knowledge acquisition prior to practical training—a feature that was positively received by participants and is in line with contemporary digital education strategies (21).

### Webinar vs. face-to-face teaching

This study principally assessed the effectiveness of webinars versus face-to-face teaching in imparting HNUS theoretical knowledge. We found that webinar teaching of HNUS theoretical skills was equivalently effective as traditional face-to-face teaching.

Our findings echo both extant studies and general trends in medical education. As with other digital education methods, webinars have recently become increasingly central in medical education, including ultrasound education, after the COVID-19 pandemic (8, 12, 13, 47, 50, 51). Learners have exhibited positive acceptance of this trend in some specialisms (13), such as ENT (12, 19). The results of our study underline that digital teaching formats are acceptable to HNUS learners and suggest they should be integrated more consistently into future training programs. Despite this positive acceptance and efficacy, educationalists should remain aware of the advantages and disadvantages of webinar teaching and use them in course designs purposively (12, 21, 50–54).

As in prior studies, webinar teaching correlated with improved knowledge, behavior, and skills comparable to in-person teaching in our data (21). This should not be surprising, as webinars enjoy certain advantages over in-person teaching. Like e-learning modules offering easy-to-access and continuously available learning opportunities (55), webinars enable more flexible learning. So-called “on-demand” or recorded webinars promote the continual reinforcement of knowledge or skills and can be used for ongoing revision. Webinars also offer the possibility of bringing together experts from different locations and time zones—synchronously, through live sessions, and asynchronously, through recordings—to discuss specialized topics (12). Leading professional societies such as EFSUMB or DEGUM have recognized these advantages, developing special topic webinars with expert participation and archiving these recordings (56).

The effectiveness of webinars for teaching theoretical skills is crucial in the context of increasing staff shortages in healthcare sectors globally. Physicians rarely have paid time to continue their professional development through in-person courses, and, understandably, employees seek to minimize the free time they spend on work-related events such as educational or continuing professional development programs. Thus, using webinars to impart theoretical knowledge can reduce time spent away from the workplace or free time devoted to work activities (12). Additionally, webinar teaching minimizes costs and time spent on travel and accommodation, providing economic and environmental benefits to employers and employees (57).

Nevertheless, webinars have limitations. Course designers must consider potential technical issues, particularly the need for a stable and fast internet connection and sufficient server capacity, as well as the challenges of purely virtual communication, which is characterized by limited group dynamics and potentially poor informal exchange among participants (54). These limitations can be addressed with careful planning and organization and with virtual breakout rooms or quiz software to enable small-group exchanges and participant interactions. Additionally, training webinar instructors is vital in implementation and quality assurance; thus, “train the trainer” modules should be the focus of research and development for webinar education (58). Certifying institutions should be increasingly involved in this development process (9, 59) and in developing standardized testing formats to evaluate the effectiveness of teaching delivered in webinars or e-learning (24). Collaboration between ultrasound didactic experts in the (further) development of assessment formats for learning outcomes could be an essential key factor in such a certification process (9).

Still, course models that include multiple smaller “webinar slots” could efficiently build relevant sonographic theory skills for practical application in person and enable more sustainable knowledge gains. Such an approach is supported by previous studies. Physicians already experienced in HNUS developed further competencies after watching lectures and live video demonstrations by HNUS experts, and they evaluated such training as effective, with most participants reporting that they felt prepared to perform the learned procedures by themselves (12). The experienced participants appreciated the time saved by avoiding traveling, though they reported missing the “hands-on” experience of in-person learning (12). Accordingly, the course in our study consisted of both teaching methods in combination: participants learned the theoretical knowledge in webinars before having the chance to discuss the lectures live with experts.

### Practical skills through digital training formats

Our findings suggest that webinars are comparably effective in imparting theoretical skills as in-person teaching. Still, it is unclear whether such webinars also promote the objective acquisition of practical ultrasound skills. One study found that “teledidactic” online seminar courses for abdominal, thoracic, and thyroid ultrasound examinations were as effective as traditional face-to-face courses and even enabled learners to outperform peers in assessing images in specific modules (namely, FAST and aorta sonography) (11, 60). Another video-based “hands-on” course facilitated significant improvements in participants’ practical skills, with the designers citing flexibility and improved access to learning materials as advantages (18, 19, 42). High-quality instructional videos are a critical component in building practical ultrasound skills through digital teaching (61), as are the “success” of the e-learning, meaning how widely adopted, high-quality, and reliable the material is according to experts, overall learner satisfaction, and the availability of qualitative learning materials (62).

In another study, students randomized into in-person and video-based teaching groups reported improvements in their self-evaluation after an HNUS practical skills course and showed good results in a practical exam (19). Similarly, another HNUS-focused study taught participants the basics of anatomy, sonoanatomy, and the setup of an ultrasound machine either through webinars (the study group) or in-person seminars (the control group). Both groups showed similar overall results in the final practical exams (19, 42).

Not all HNUS education researchers have unanimously approved webinar teaching for practical ultrasound skills. Everad et al. guided webinar participants in moving an ultrasound probe dummies while they watched a video of the ultrasound image (42). This method was sufficient to teach the examination of most regions relevant to HNUS. Still, structures like the pulsatile common carotid artery or the omohyoid muscle requiring dexterous movement of the ultrasound probe for identification were poorly examined, with the seminars lacking real-time demonstration and the necessary visual-tactile feedback to achieve complicated imaging (9, 42). Nevertheless, the authors noted that certain aspects of practical teaching being moved to webinars could benefit teaching efficiency and, by extension, final student outcomes.

### Limitations

The present study has several limitations that must be considered when interpreting the results. First, participants were not randomly assigned to the groups, as allocation was based on course registration. This introduces a risk of selection bias since participants may have chosen their course format (webinar vs. face-to-face) based on personal preferences or availability. While this reflects real-world conditions in certified postgraduate training programs, it may limit the internal validity and generalizability of the findings. Future studies should aim to implement randomized group allocation to strengthen causal inferences. Second, no pre-and post-test for purely theoretical competencies was conducted, making direct comparisons of knowledge gains between the groups difficult. Third, personal factors such as clinical experience and participant motivation could have influenced the study outcomes. These variables were not controlled for in this study. Additionally, the courses were led by different instructors, which could have introduced variability in the teaching methodology. Additionally, there was a significant imbalance between the groups: the study group had 1 week between the webinar and the practical part, whereas the control group had the theoretical and practical parts on two consecutive days. Moreover, participants in the study group had access to the recorded webinar content, potentially giving them an advantage in preparing for the theory test and practical day. So far, the study only evaluated short-term outcomes, such as immediate post-course assessments. It did not assess long-term knowledge retention or the effectiveness of each teaching method in improving clinical practice over time. Further potential influencing factors, such as technological affinity and access to technical resources, were also not measured. Finally, the study was conducted solely in the field of otorhinolaryngology. Therefore, the generalizability of the results to other medical specialties or types of courses is unclear. Future studies should address these limitations by employing randomized designs, including pre-and post-tests for theoretical knowledge, and examining the long-term effects of different teaching methods.

## Conclusion

Despite the limitations discussed, including non-randomized group allocation, the study provides evidence that digitally supported teaching concepts in webinar teaching can achieve equivalent theoretical competence levels in head and neck ultrasound as traditional face-to-face teaching concepts. The data collected in this study, including the positive evaluations of the digitally supported teaching concept and attitudes toward digitalization, underscore the potential of a blended learning-based sonography education. This teaching approach should feature in accreditation and certification processes in the future.

## Data Availability

The raw data supporting the conclusions of this article will be made available by the authors, without undue reservation.
