# Supplementary material for: Ultrasound education in the digital era: face-to-face vs. webinar-teaching of head and neck ultrasound theory—a prospective multi-center study
Source: Front Med (Lausanne). 2025 May 9;12:1506260. doi: 10.3389/fmed.2025.1506260 (PMC12098340; doi:10.3389/fmed.2025.1506260)
Supplement: Supplementary file 1 [file Data_Sheet_1.pdf]

Supplement 1 Theory-Test^{post} structure and example questions

Number	Topic	Question type	Points
1	Lymph nodes	Kprim	4
2		B	4
3		A	1
4		A	1
5		Aneg	1
6		Aneg	1
7	Pathologies of the Neck	R + free text	10
8		Aneg	1
9		Aneg	1
10		A	1
11		A	1
12		Aneg + free text	2
13		Aneg	1
14		R + free text	9
15	Salivary glands	Kprim	4
16		R + free text	8
17		Kprim	4
Total points			

Topic: Lymph Nodes

1) Which of the following statements about the ultrasound findings of a patient with neck pain and multiple palpable nodes for about 3 weeks are correct, and which are incorrect?
(Question type: Kprim)

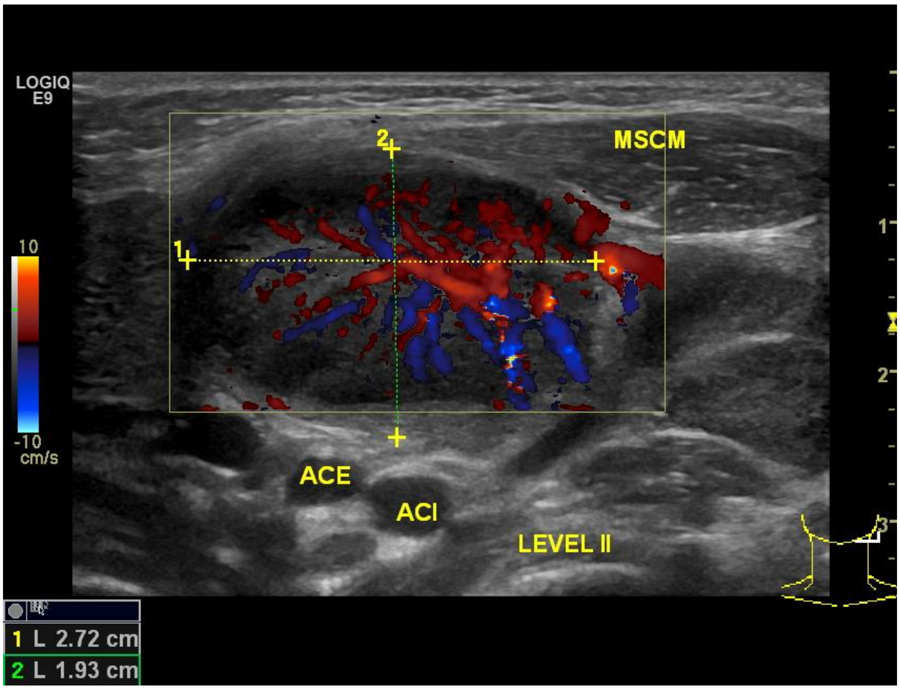

a) It shows the diffuse vascularisation typical of a malignant finding.

☐ correct ☐ incorrect

b) The vascularisation pattern in doppler sonography corresponds most likely to a centrally branching type.

☐ correct ☐ incorrect

c) The rather oval shape of the lymph node suggests a benign process.

☐ correct ☐ incorrect

d) A mediolateral diameter of 2.72 cm is to be considered physiological.

☐ correct ☐ incorrect

J.Weimer; M.Rink., L.Symeou, M.Brandt, N.Mansour, J.Künzel

2) Which of the images shown here on the left *most likely shows the sonomorphological appearance of:*
(Question type: B)

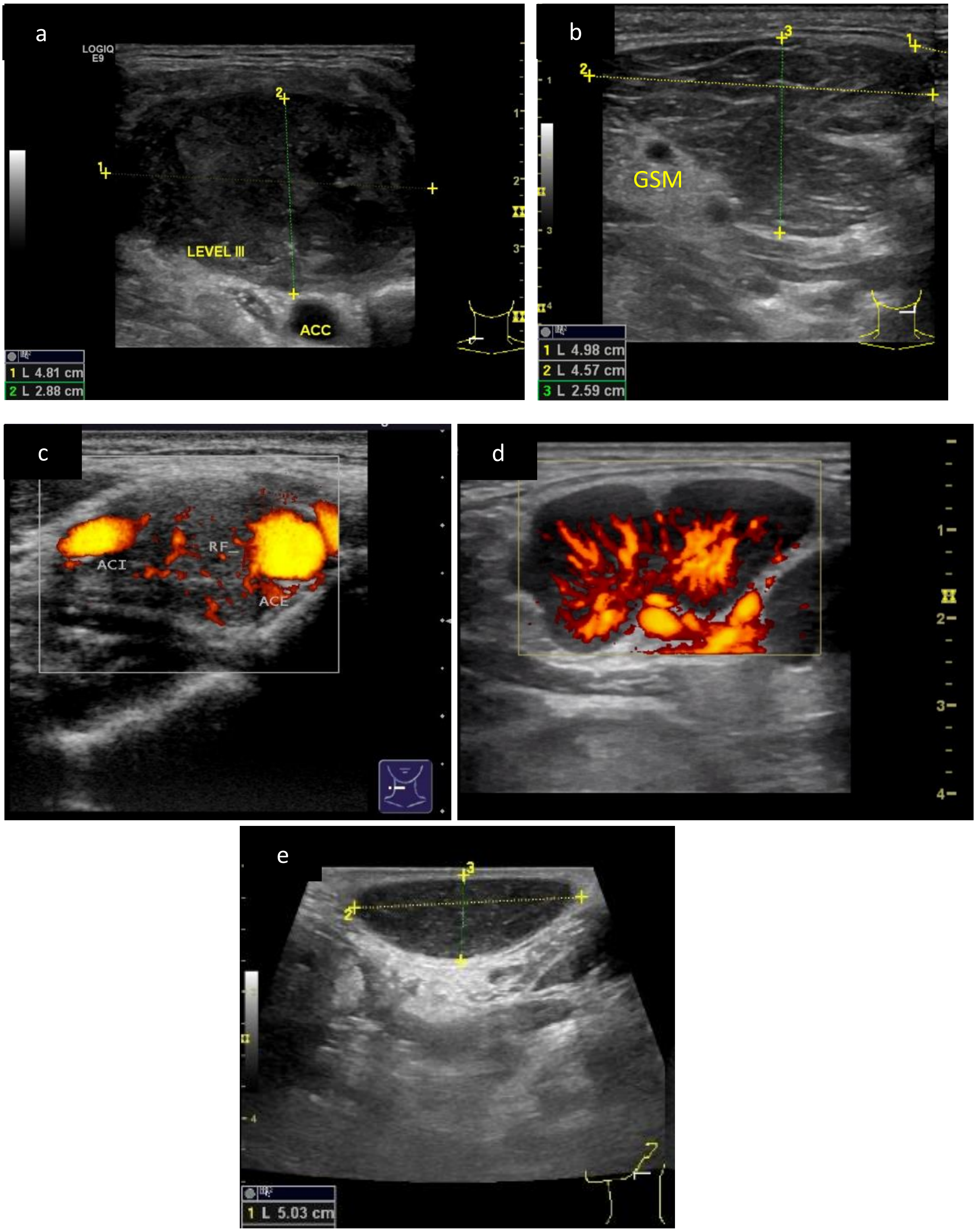

J.Weimer; M.Rink., L.Symeou, M.Brandt, N.Mansour, J.Künzel

2. 1. A lymph node suspicious for malignancy<br><br>(a) (b) (c) (d) (e)	*2.2. A reactive lymph node*<br><br>(a) (b) (c) (d) (e)
2.3. A lipoma<br><br>(a) (b) (c) (d) (e)	*2.4. A paraganglioma*<br><br>(a) (b) (c) (d) (e)

3) Which of the following statements about lymph node sonography is correct (**Question type: A**)?

- For the evaluation of cervical lymph nodes, native CT of the neck is superior to sonography.
- Physiologically, round lymph nodes are never found cervically.
- The assessment of the vascularisation pattern plays a subordinate role in the assessment of dignity.
- Multiparametric ultrasound (elastography, contrast-enhanced ultrasound) offers high potential to improve dignity assessment.
- Ultrasound-guided biopsy is contraindicated in suspected malignantly altered lymph nodes.

4) Which of the following findings is most consistent with the presence of lymphoma? **(Question type: A)**

  - "In level II on the right, a single oval node with a clearly visible hilum sign, sharply demarcated from the surroundings, diameter of the long axis at 8 mm"
  - "In level V on the left, large (4x2 cm), poorly demarcated node with inhomogeneous echogenicity, pronounced peripheral vascularisation, central suspicion of liquid parts"
  - "In level III on the right, a conglomerate of a total of 4 round lymph nodes with homogeneous echogenicity, partially hilar, partially peripheral vascularisation. All nodes of the conglomerate with a diameter >10 mm."
  - "In level Ia a single round lymph node with a diameter of about 4 mm, well demarcated from the surroundings, no vascularisation demonstrable."

5) Which of the following answers regarding lymph node sonography is most likely incorrect? **(Question type: Aneg)**

- Typically, in sonography, an oval, sharply defined shape is observed.
- Typically, in sonography, a hypoechoic hilum is observed.
- For reliable identification, displaying in a second plane helps.
- An MRI for follow-up of enlarged lymph nodes is not necessarily required.

6) Which statement about the image below is **least accurate? (Question type: Aneg)**

- The finding is in the deep lobe of the left parotid gland.
- Duplex sonography should be performed.
- The typical morphology of a lymph node is not recognisable.
- Certain areas may already be necrotic.
- Histological confirmation can be obtained through core biopsy.

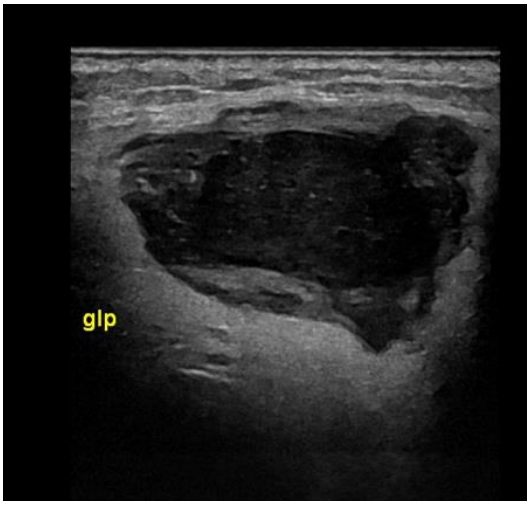

Topic: Pathologies of the Neck (excluding lymph nodes)

7) For the following patient cases, choose one suspected diagnosis and at least one possible differential diagnosis that could characterise the sonographic findings **(Question type: R)**

A) Dermoid	J) Neck abscess
B) Lipoma	K) Atheroma
C) Neurofibroma	L) Branchial cleft cyst
D) Ectopic thyroid tissue	M) Salivary gland tumour
E) thyroglossal duct cyst	N) Saliva fistula/ seroma
F) Hemangioma and lymphangioma	O) Sialolithiasis
G) Paraganglioma	P) Thyroid nodule
H) Neurinoma	Q) Nasal bone fracture
I) Hematoma and seroma	R) Furuncle

A) An 11-year-old girl complains that her ski helmet pressed behind her left ear at the end of a ski day. The mother noticed redness and swelling posterior to the left earlobe. Treatment with drawing salve has not improved so far. There are no pre-existing conditions or previous surgeries.

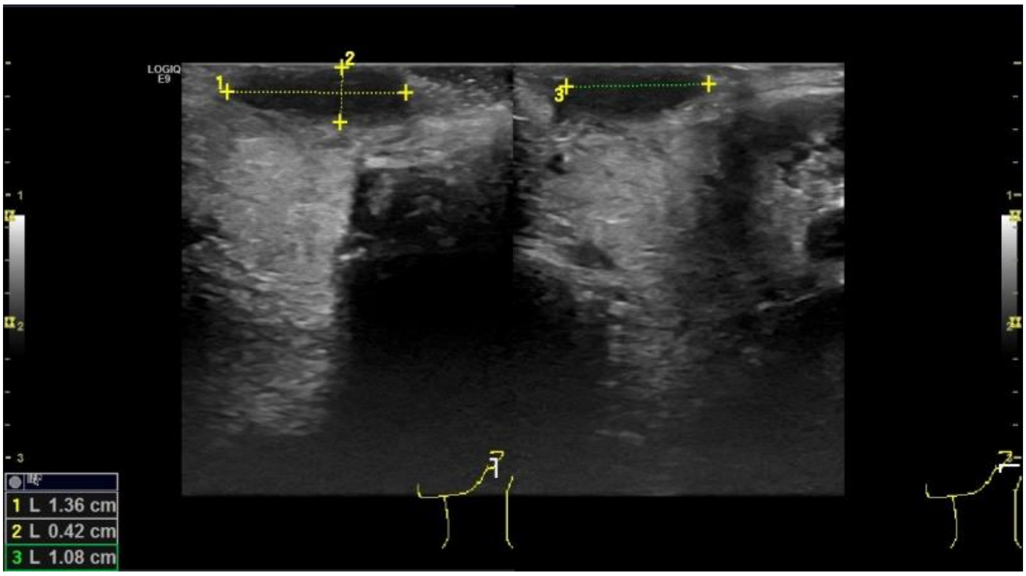

Diagnosis: ____________________Differential diagnosis: __________________

B) A 30-year-old woman with an indolent swelling in the middle of her neck for 3 months, stable in size. Appeared after an upper respiratory infection with tonsillitis. No redness or warmth. No treatment has been given so far.

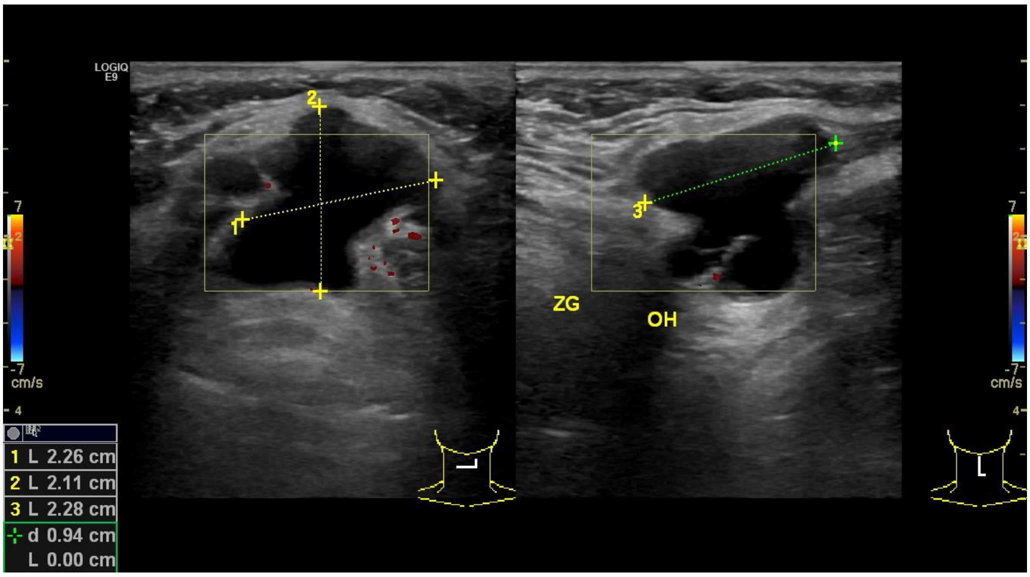

Diagnosis: ____________________Differential diagnosis: __________________

J.Weimer; M.Rink., L.Symeou, M.Brandt, N.Mansour, J.Künzel

C) A 60-year-old man with a history of prostate cancer is concerned about an indolent swelling, stable in size, at the angle of his jaw on the right side, which he noticed 6 months ago.

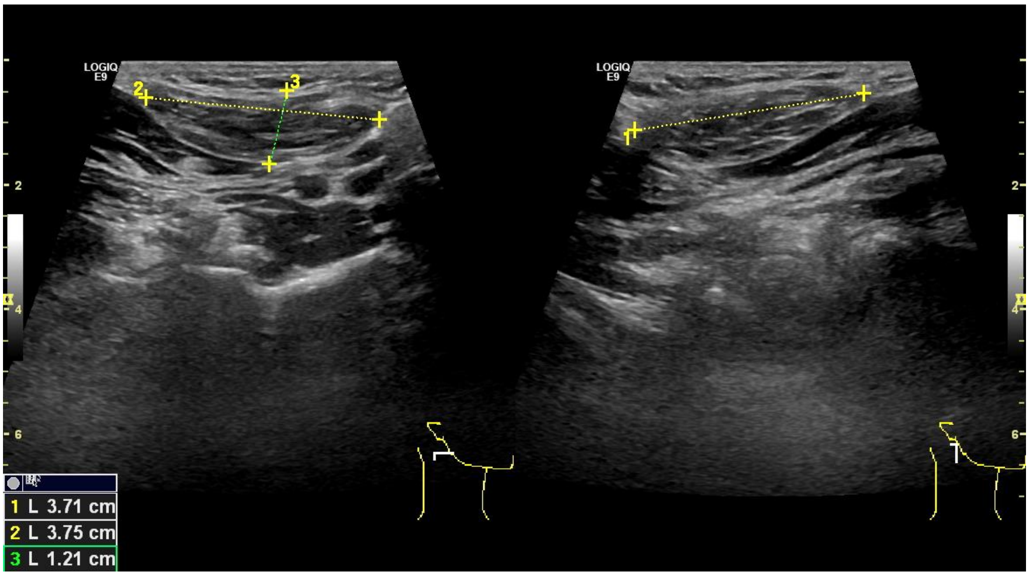

Diagnosis: ____________________Differential diagnosis: __________________

D) A 40-year-old man with an incidental finding on MRI. Upon questioning, he reports occasional swallowing difficulties. Laryngoscopic examination reveals reduced mobility of the right vocal cord. No B symptoms known.

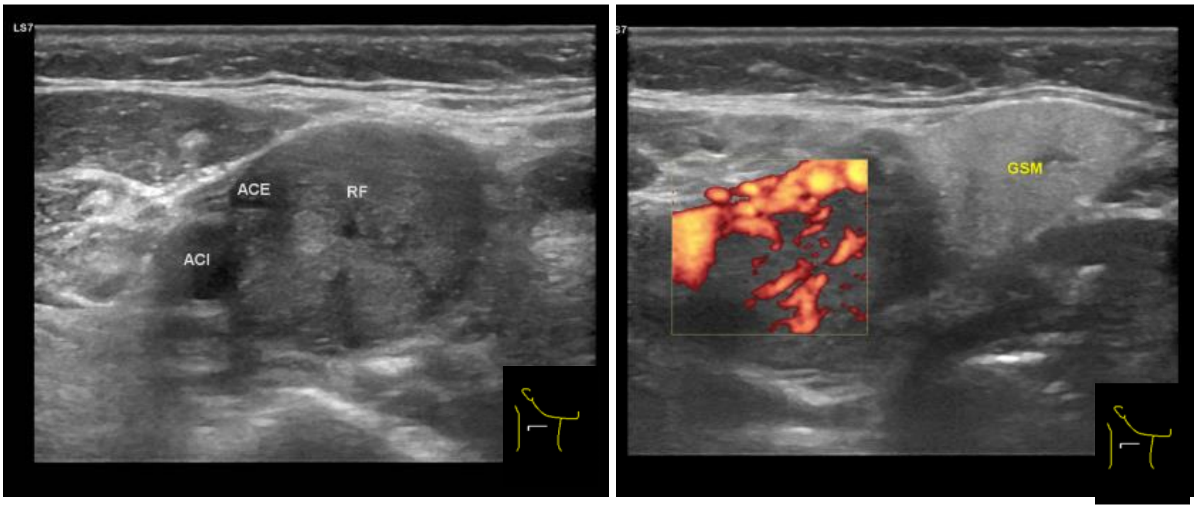

Diagnosis: ____________________Differential diagnosis: __________________

J.Weimer; M.Rink., L.Symeou, M.Brandt, N.Mansour, J.Künzel

E) A 47-year-old man reports a slightly size-progressing swelling pre-auricularly on the left and moderate pain since the day before yesterday. He underwent a partial parotidectomy on the left two days ago.

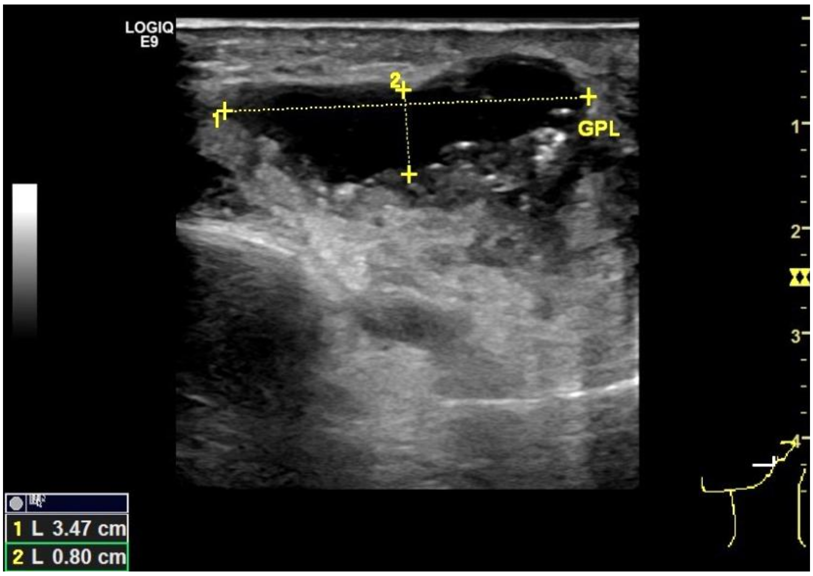

Diagnosis: ____________________Differential diagnosis: __________________

8) Which of the following answers regarding a thyroglossal duct cyst is most likely incorrect? **(Question type: Aneg)**

- The formation can be attached to the hyoid bone.
- Typically, in sonography, there is distal acoustic enhancement.
- Patients often report fluctuations in size.
- The formation can extend below or above the hyoid bone.

9) Which of the following answers regarding a lipoma is **most likely incorrect? (Question type: Aneg)**

- The sonographic appearance can resemble muscle.
- The formation is located subcutaneously or in deeper neck soft tissues.
- The structure is hard in consistency during palpation.
- Hypervascularity is not expected on ultrasound.

10) Which of the following answers regarding a carotid paraganglioma is **most likely correct? (Question type: A)**

- o A core biopsy should be performed for diagnostic confirmation.
- o The sonographic image often shows a splaying of the internal and external carotid (Lyre-sign).
- o A carotid paraganglioma is clearly differentiated from a vagal paraganglioma by its location.
- o The duplex image shows predominantly peripheral vascularisation.

11) Which of the following answers regarding a neck abscess is **most likely correct? (Question type: A)**

- o Sonographically, it usually shows a sharply demarcated structure.
- o The vascularisation directed from central to peripheral is typical for an abscess.
- o Surrounding tissue often shows an inflammatory reaction.
- o Due to the liquid components, a distal shadowing is often observed.

12) Which of the following answers regarding the findings of a postoperative sonography is **most likely incorrect? (Question type: Aneg)**

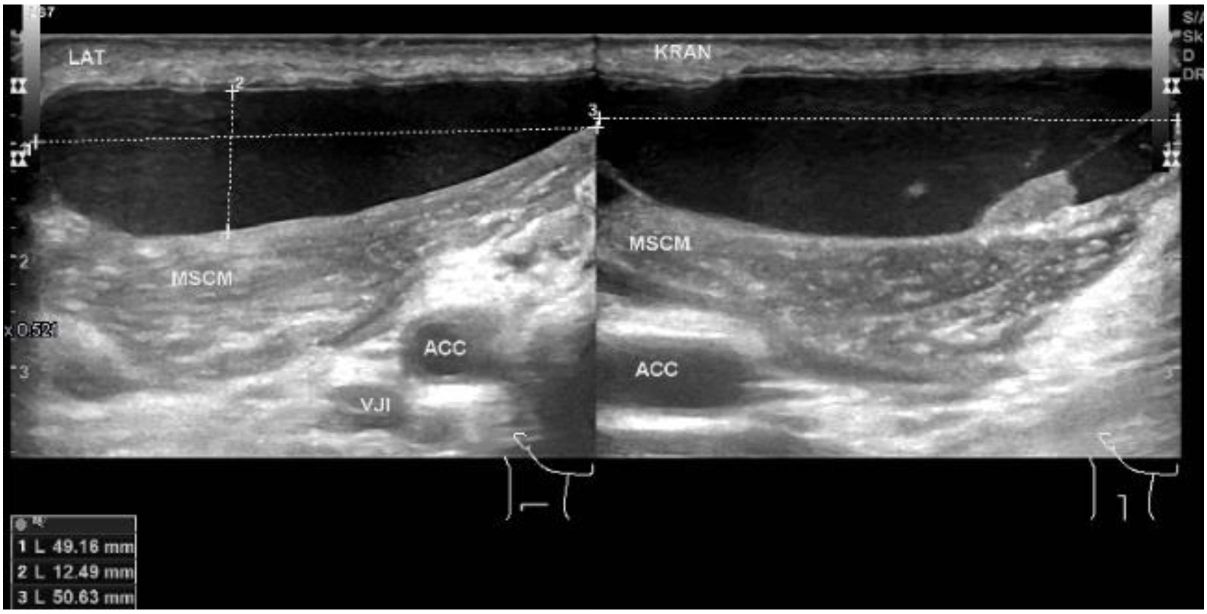

- The measured mass is mostly anechoic.
- No vascularisation would be expected in a duplex sonography.
- The sonogram shows a superficially located mass.
- A sonographically controlled core biopsy would be advisable.

What is your diagnosis? ____

13) Which of the following answers regarding a bland atheroma is **most likely incorrect? (Question type: Aneg)**

- It is usually located in the subcutaneous tissue.
- Sonographically, it can appear hypoechoic, homogeneous with partial distal acoustic enhancement.
- A colour duplex image often shows diffuse vascularisation.
- In case of size progression, surgical removal (excision) should be considered.

14) For the following patient cases, select one suspected diagnosis and at least one possible differential diagnosis that could characterise the sonographic findings **(Question type: R).**

A) Acute sinusitis	I) Phlegmon
B) Chronic sinusitis	J) Abscess
C) Cyst	K) Malignant tumour
D) Branchial cleft cyst	L) Benign tumour
E) Polyp	M) Dislocated nasal bone fracture
F) Mucosal swelling	N) Dislocated zygomatic arch fracture
G) Vascular malformation	O) Mandible fracture
H) Masseter hypertrophy	P) Orbital floor fracture
	Q) HPV positive lymph node metastasis

A) A 15-year-old boy presents to the emergency room after experiencing an elbow trauma during football training, with a painful, swollen nose.

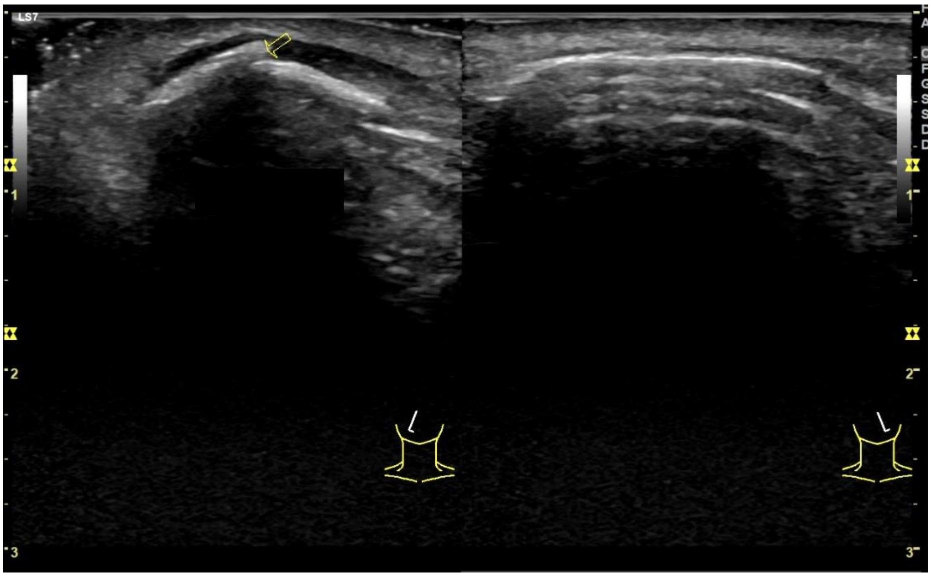

Diagnosis: ____________________Differential diagnosis: __________________

B) A 64-year-old patient presents with a painless swelling on the right side of the lower jaw at the clinic. The mass is poorly movable and firm. An ultrasound examination is conducted.

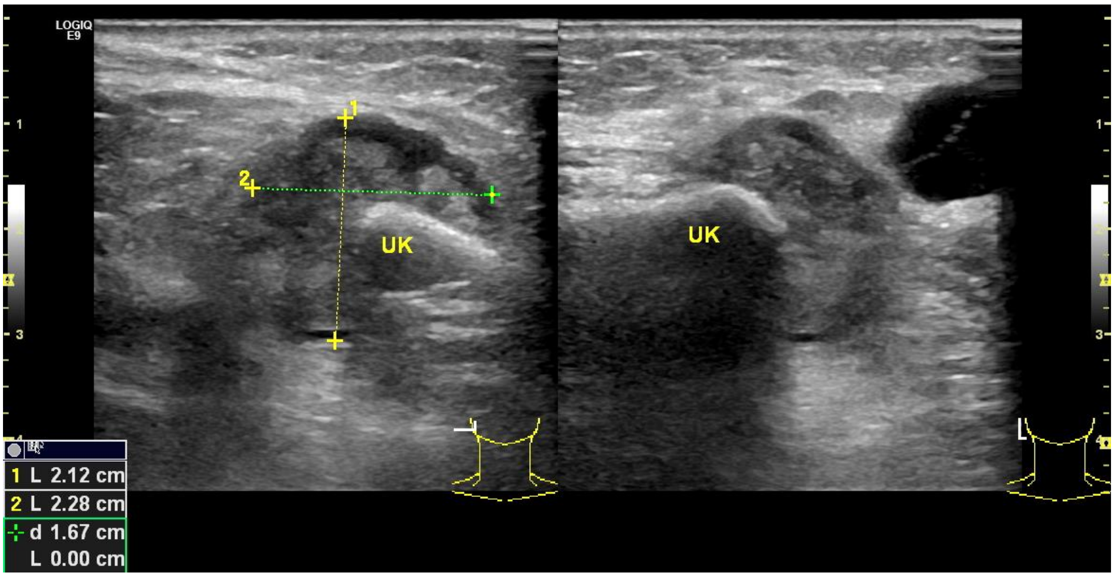

Diagnosis: ____________________Differential diagnosis: __________________

What further examination would you order? __________________________

J.Weimer; M.Rink., L.Symeou, M.Brandt, N.Mansour, J.Künzel

C) A 54-year-old patient with a history of nicotine abuse presents with a swelling in the right parotid region that has been constant in size for 3 months. An ultrasound examination is conducted.

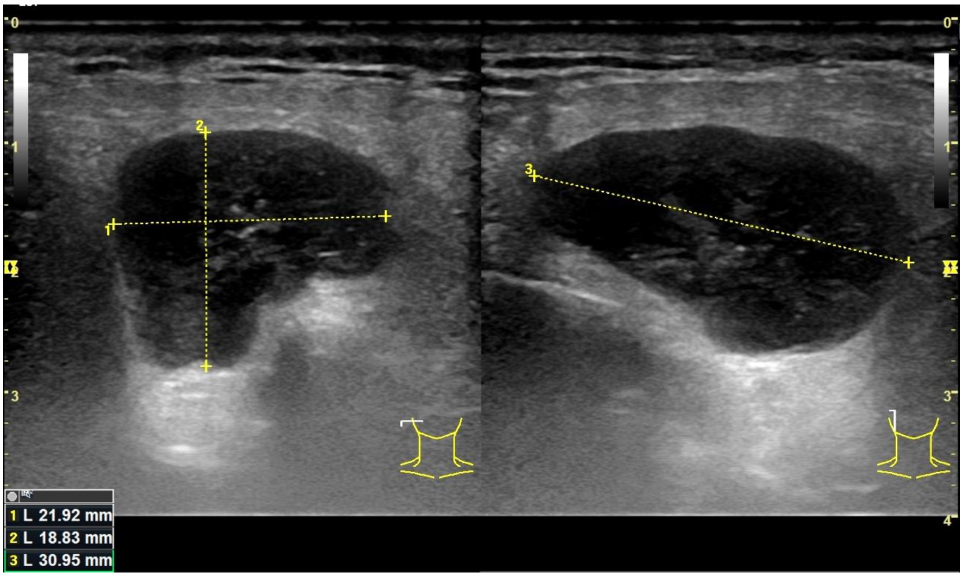

Diagnosis: ____________________Differential diagnosis: __________________

D) A 35-year-old patient with a total of 5 pack-years presents with a right-sided neck swelling that has been present for 3 weeks. No abnormalities were found on a preliminary ENT-specific examination.

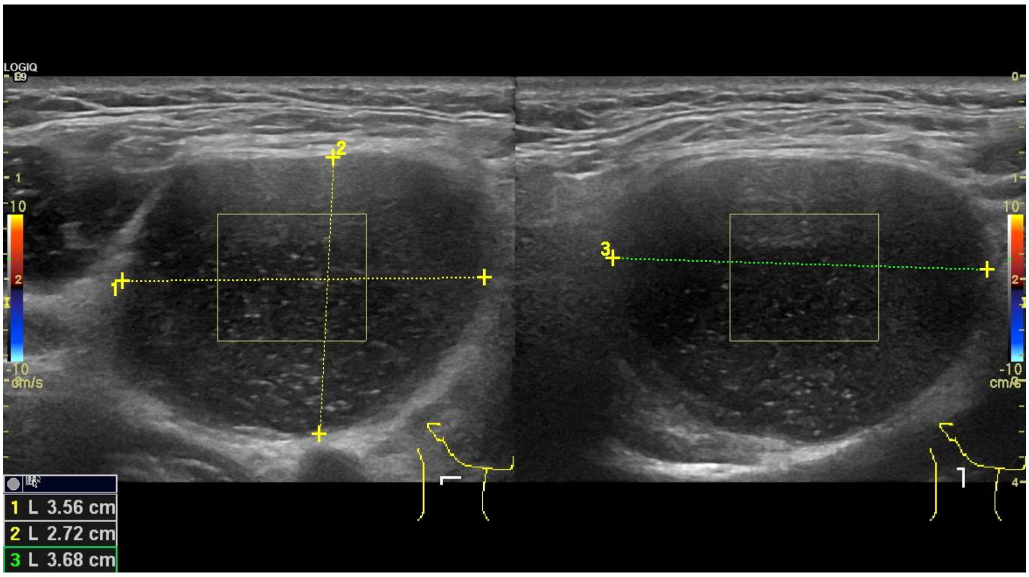

Diagnosis: ____________________Differential diagnosis: __________________

Topic: Salivary Glands

15) A 55-year-old patient presents with recurrent swellings of the left cheek that occur with eating. No pain is present. What findings can you typically expect in the sonography? **(Question type: Kprim)**

a) Echogenic reflex in the course of the Stensen's duct

☐ correct ☐ incorrect

b) Completely atrophied, scarred, and barely distinguishable gland

☐ correct ☐ incorrect

c) Proximally dilated duct

☐ correct ☐ incorrect

d) Distal acoustic shadowing behind the echogenic reflex

☐ correct ☐ incorrect

16) Assign the most likely suspected diagnosis to the images and name a differential diagnosis **(Question type: R).**

A) Cyst	G) Küttner tumour
B) Sjögren's syndrome	H) Abscess
C) Salivary gland carcinoma	I) Ranula
D) Benign tumour	J) Lymphangioma
E) Stensen's duct stenosis	K) Lymphoma
F) Sialolithiasis	L) Lymph node metastasis

A) A 50-year-old patient presents with a palpable, painless, rapidly progressing swelling of the right parotid gland.

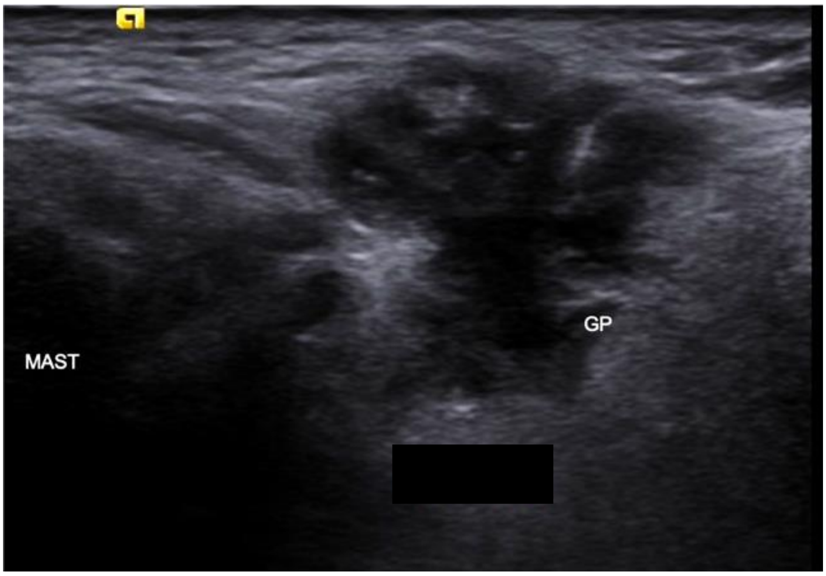

Diagnosis: ____________________Differential diagnosis: __________________

B) A 50-year-old female patient presents with a palpable, painless swelling of the right parotid gland.

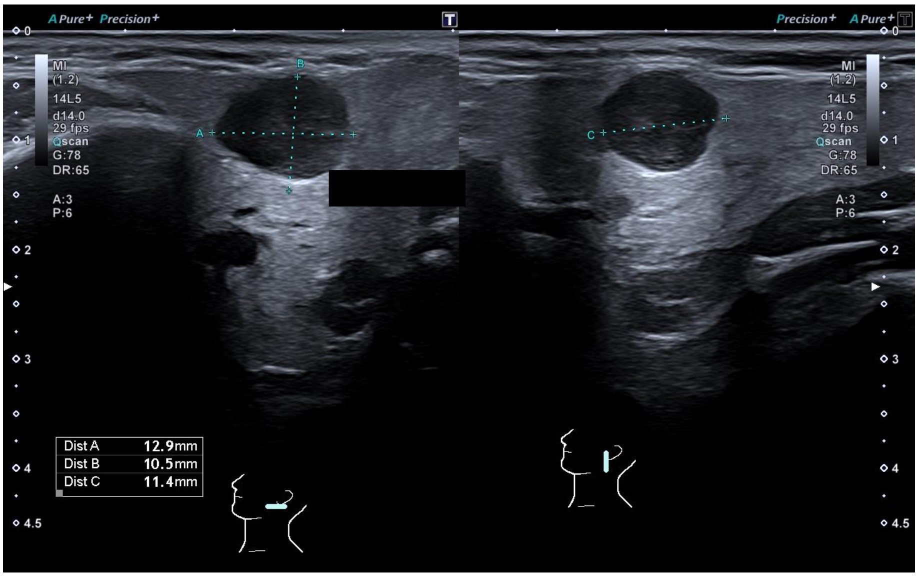

Diagnosis: ____________________Differential diagnosis: __________________

J.Weimer; M.Rink., L.Symeou, M.Brandt, N.Mansour, J.Künzel

C) A 2-year-old child is presented with a soft swelling of the floor of the mouth.

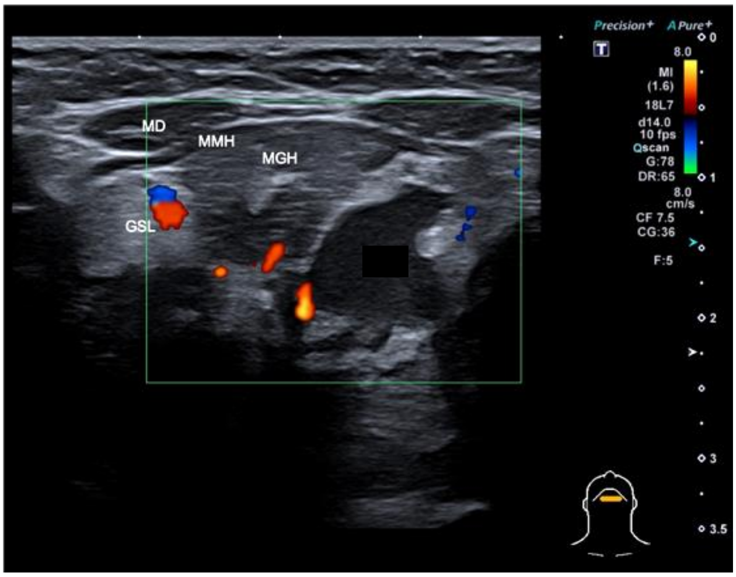

Diagnosis: ____________________Differential diagnosis: __________________

D) A 30-year-old patient reports recurrent swellings of the left cheek.

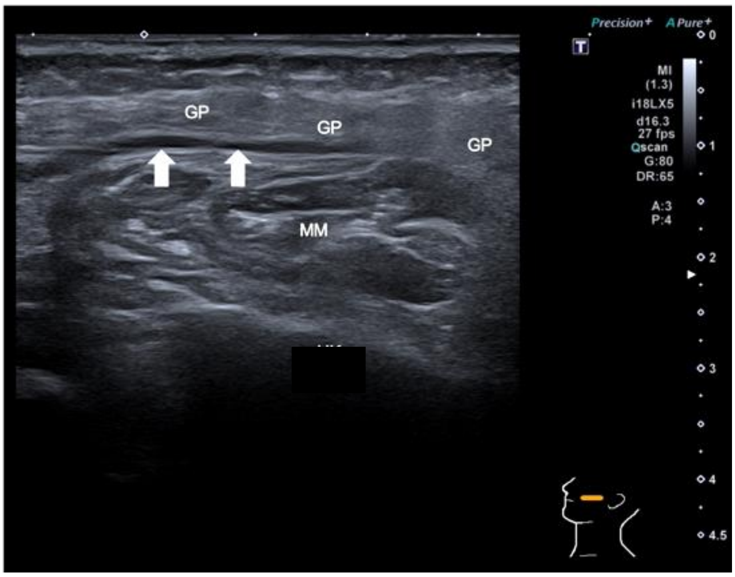

Diagnosis: ____________________Differential diagnosis: __________________

17) A 70-year-old patient has a carcinoma of the left parotid gland. What sonographic findings can be present? **(Question type: Kprim)**

a) Sharp tumour margins

☐ correct ☐ incorrect

b) Enlarged and numerically increased lymph nodes in levels II and III on the left

☐ correct ☐ incorrect

c) Infiltration into the masseter muscle

☐ correct ☐ incorrect

d) Peripheral vascularisation in colour doppler sonography

☐ correct ☐ incorrect
