# Supplementary material for: Ultrasound education in the digital era: face-to-face vs. webinar-teaching of head and neck ultrasound theory—a prospective multi-center study
Source: Front Med (Lausanne). 2025 May 9;12:1506260. doi: 10.3389/fmed.2025.1506260 (PMC12098340; doi:10.3389/fmed.2025.1506260)
Supplement: Supplementary file 2 [file Data_Sheet_2.pdf]

Supplement 2 Recruitment process and inclusion of participants in the study

	Center 1	Center 2	Center 3	
	Study group	Study group		Validation group
Registered course participants	40	22	31	53
Attended the course	37	22	28	50
No consent to participate in the study	4	3	10	3
Test not completely processed	2	2	5	0
Included in analysis	31	30		47
