# Supplementary material for: Ultrasound education in the digital era: face-to-face vs. webinar-teaching of head and neck ultrasound theory—a prospective multi-center study
Source: Front Med (Lausanne). 2025 May 9;12:1506260. doi: 10.3389/fmed.2025.1506260 (PMC12098340; doi:10.3389/fmed.2025.1506260)
Supplement: Supplementary file 3 [file Data_Sheet_3.pdf]

Supplement 3 Subjective competence assessment and subjective attitude assessment for digitally supported ultrasound courses before and after completing a DEGUM-certified head and neck advanced course; 7-point Likert scale (1= strongly disagree with the statement; 7= strongly agree with the statement)

T1: time point Evaluation^{pre}, upon enrollment, T2: time point Evaluation^{post}, at the end of the course, T1-T2: between time points T1 and T2, SD: standard deviation

	Control group T1	Study group T1	P-value	Control group T2	Study group T2	P-value	Delta Control group<br>p-Value T1-T2	Delta Study group<br>p-value T1-T2	Detla p-value
**Subjective competency assessment**									
	Mean ± SD	Mean ± SD		Mean ± SD	Mean ± SD		Mean ± SD<br>p-Value T1-T2	Mean ± SD<br>p-Value T1-T2	
Basic competences overall	5.06 ± 1.06	4.93 ± 0.75	0.59	5.82 ± 0.68	5.7 ± 0.57	0.44	0.75 ± 0.83<br>0.003	0.77 ± 0.92<br><0.001	0.92
Anatomy	5.68 ± 0.8	5.1 ± 0.75	0.007	6.0 ± 0.7	5.81 ± 0.6	0.25	0.28 ± 0.61<br>0.12	0.71 ± 0.94<br><0.001	0.04
Sonoanatomy	5.0 ± 1.08	4.81 ± 0.87	0.47	5.8 ± 0.61	5.9 ± 0.75	0.56	0.8 ± 0.96<br>0.002	1.1 ± 1.11<br><0.001	0.29

Physical basics	4.56 ± 1.5	4.13 ± 1.23	0.25	5.17 ± 1.09	5.0 ± 1.18	0.57	0.68 ± 1.11	0.87 ± 1.41	0.57
							0.1	0.006	
Understanding image formation	4.96 ± 1.31	4.68 ± 1.11	0.39	5.3 ± 1.09	5.23 ± 1.2	0.8	0.32 ± 1.15	0.55 ± 1.36	0.5
							0.31	0.07	
Ultrasound image orientation	5.4 ± 1.41	5.45 ± 1.06	0.88	6.1 ± 0.85	6.03 ± 0.71	0.74	0.68 ± 1.49	0.58 ± 1.31	0.79
							0.04	0.01	
Transducer handling	5.48 ± 1.36	5.55 ± 1.09	0.84	6.27 ± 0.64	6.03 ± 0.66	0.16	0.76 ± 1.33	0.48 ± 1.21	0.43
							0.01	0.04	
Image optimisation	4.24 ± 1.64	4.52 ± 1.21	0.49	6.03 ± 0.89	5.61 ± 0.76	0.05	1.76 ± 1.54	1.1 ± 1.27	0.09
							<0.001	<0.001	
Artefacts	4.64 ± 1.29	4.42 ± 1.21	0.51	5.6 ± 1.04	5.39 ± 0.72	0.36	1.0 ± 1.12	0.97 ± 1.28	0.92
							0.004	<0.001	
Patient guidance	5.6 ± 1.41	5.68 ± 1.19	0.83	6.13 ± 0.78	6.29 ± 0.64	0.39	0.48 ± 1.19	0.61 ± 1.26	0.69
							0.1	0.02	
Overall pathological competences	4.58 ± 1.14	4.53 ± 0.81	0.86	5.77 ± 0.64	5.63 ± 0.51	0.34	1.19 ± 0.99	1.1 ± 0.86	0.7
							<0.001	<0.001	

Sonopathology in general	5.12 ± 1.01	4.94 ± 1.03	0.5	5.93 ± 0.58	5.77 ± 0.56	0.28	0.76 ± 0.83	0.84 ± 1.1	0.76
							0.001	<0.001	
Soft tissues of the neck	4.88 ± 1.27	5.03 ± 1.05	0.63	5.93 ± 0.64	5.81 ± 0.6	0.43	1.04 ± 1.14	0.77 ± 1.12	0.38
							<0.001	<0.001	
Diseases of the salivary gland	4.8 ± 1.35	4.68 ± 1.05	0.71	6.07 ± 0.64	5.71 ± 0.69	0.04	1.24 ± 1.17	1.03 ± 1.02	0.49
							<0.001	<0.001	
Lymph nodes	5.32 ± 1.25	5.19 ± 0.98	0.68	6.13 ± 0.63	5.9 ± 0.65	0.17	0.8 ± 1.08	0.71 ± 1.01	0.75
							0.005	<0.001	
Paranasal sinuses	3.72 ± 1.7	3.65 ± 1.66	0.87	5.3 ± 0.92	4.94 ± 0.89	0.12	1.64 ± 1.66	1.29 ± 1.81	0.45
							<0.001	<0.001	
Bony structures of the face	3.64 ± 1.91	3.71 ± 1.44	0.88	5.27 ± 1.2	5.65 ± 0.84	0.16	1.68 ± 1.7	1.94 ± 1.53	0.56
							<0.001	<0.001	
**Subjective attitude assessment**									
Future prospects	5.25± 1.06	5.70 ±0.61	0.05	5.56 ±0.75	6.00 ± 0.45	0.009	0.53 ± 0.88	0.30 ± 0.49	0.27
							0.23	0.03	

Offer digital teaching media	4.17 ± 1.58	5.4 ± 1.07	0.002	5.04 ± 1.62	6.32 ± 0.83	0.0005	1.00 ± 1.71<br>0.06	0.93 ± 1.26<br><0.001	0.86
Expansion of digital US media	5.68 ± 1.68	5.71 ± 1.3	0.94	6.04 ± 1.4	6.1 ± 1.14	0.86	0.74 ± 1.6<br>0.41	0.39 ± 1.38<br>0.22	0.4
Digital analogue ratio	3.6 ± 1.29	4.03 ± 1.17	0.2	4.04 ± 1.55	3.94 ± 1.37	0.79	0.46 ± 1.18<br>0.27	-0.1 ± 1.3<br>0.77	0.1
Future of blended learning	5.44 ± 1.64	6.16 ± 1.21	0.07	5.75 ± 1.4	6.39 ± 0.84	0.04	0.63 ± 1.72<br>0.47	0.23 ± 1.06<br>0.4	0.32
Expansion of blended learning	5.64 ± 1.35	6.1 ± 1.22	0.2	5.86 ± 1.3	6.36 ± 0.84	0.09	0.54 ± 0.88<br>0.55	0.26 ± 0.93<br>0.34	0.25
Integration of case studies	5.88 ± 1.39	6.39 ± 0.88	0.12	6.32 ± 1.19	6.42 ± 0.92	0.73	0.67 ± 1.4<br>0.22	0.03 ± 0.8<br>0.89	0.06
Webinar for Continuing Medical Education	5.8 ± 1.53	6.19 ± 0.95	0.27	6.04 ± 1.14	6.42 ± 0.72	0.15	0.3 ± 1.15<br>0.53	0.23 ± 0.81<br>0.29	0.78
Webinar teaching theoretical knowledge	5.8 ± 1.53	6.19 ± 0.95	0.27	6.2 ± 0.87	6.71 ± 0.46	0.01	0.35 ± 1.19<br>0.56	0.45 ± 1.12<br>0.05	0.75

Webinar recordings before follow-up	5.92 ± 1.35	6.26 ± 0.97	0.3	6.28 ± 0.84	6.61 ± 0.56	0.1	0.52 ± 1.2<br>0.27	0.36 ± 0.84<br>0.08	0.57
Webinar attendance ratio	4.32 ± 1.38	4.48 ± 1.24	0.65	4.36 ± 1.41	4.77 ± 0.92	0.21	0.22 ± 1.28<br>0.92	0.29 ± 1.1<br>0.3	0.83
