# Supplementary material for: Ultrasound education in the digital era: face-to-face vs. webinar-teaching of head and neck ultrasound theory—a prospective multi-center study
Source: Front Med (Lausanne). 2025 May 9;12:1506260. doi: 10.3389/fmed.2025.1506260 (PMC12098340; doi:10.3389/fmed.2025.1506260)
Supplement: Supplementary file 4 [file Data_Sheet_4.pdf]

Supplement 4 Course and webinar evaluation (T2)

	**Control-group (mean±SD)**	**Study-group (mean±SD)**	**p-value**
**Overall course evaluation**	6.43 ± 0.65	6.51 ± 0.6	0.65
Course concept	6.4 ± 0.81	6.45 ± 0.72	0.79
Use of time	6.3 ± 0.84	6.45 ± 0.81	0.48
Theory to practice ratio	6.27 ± 0.94	6.58 ± 0.67	0.14
Live lectures	6.43 ± 0.77	6.39 ± 0.76	0.81
Functionality of technology in face-to-face	6.7 ± 0.6	6.68 ± 0.54	0.88
Interaction in classroom teaching	6.5 ± 0.63	6.48 ± 0.77	0.93
**Overall webinar evaluation**	xxxxx	6.26 ± 0.73	xxxxx
Webinar lectures	xxxxx	6.19 ± 0.75	xxxxx
Functionality of technology in Webinar	xxxxx	6.52 ± 0.63	xxxxx
Webinar interaction	xxxxx	6.07 ± 1.15	xxxxx
Recorded webinar videos benefits	xxxxx	5.16 ± 2.15	xxxxx
