# Supplementary material for: Ultrasound education in the digital era: face-to-face vs. webinar-teaching of head and neck ultrasound theory—a prospective multi-center study
Source: Front Med (Lausanne). 2025 May 9;12:1506260. doi: 10.3389/fmed.2025.1506260 (PMC12098340; doi:10.3389/fmed.2025.1506260)
Supplement: Supplementary file 5 [file Data_Sheet_5.pdf]

Supplement 5 Results of the Theory-Test^{Post} of the control group and study group, including presentation of the question type

Topic and Number		Question type	Max points	Control-group (mean±SD)	Study-group (mean±SD)	p-value
**Total Score Theory-Test^{post}**			54	36.67 ± 4.74	38.1 ±5.72	0.541
**Score Pathologies Lymph nodes**			12	9.23 ± 2.1	9.74 ± 1.46	0.275
A 1		Kprim	4	3.37 ± 0.72	3.26 ± 0.73	0.56
A 2		B	4	2.73 ± 1.2	2.87 ± 1.02	0.63
A 3		A	1	0.9 ± 0.31	0.97 ± 0.18	0.3
A 4		A	1	0.73 ± 0.45	0.9 ± 0.3	0.09
A 5		Aneg	1	0.87 ± 0.35	0.9 ± 0.3	0.66
A 6		Aneg	1	0.63 ± 0.49	0.84 ± 0.37	0.07
**Score Pathologies- soft tissues of the neck**			26	16.87 ± 2.79	17.52± 3.17	0.4
A 7		R + Free-text	10	6.43 ± 1.36	6.71 ± 1.49	0.45
A7a			*2*	1.53 ± 0.57	1.68 ± 0.54	0.32
A7b			*2*	1.27 ± 0.52	1.13 ± 0.43	0.26
A7c			*2*	1.23 ± 0.5	1.07 ± 0.77	0.32
A7d			*2*	1.13 ± 0.78	1.32 ± 0.7	0.32
A7e			*2*	1.27 ± 0.69	1.52 ± 0.51	0.12
A 8		Aneg	1	0.03 ± 0.18	0.13 ± 0.34	0.18
A 9		Aneg	1	0.93 ± 0.25	0.81 ± 0.4	0.15
A 10		A	1	0.8 ± 0.41	0.74 ± 0.45	0.6
A 11		A	1	0.87 ± 0.35	0.9 ± 0.3	0.66
Ges_A 12		Aneg + Free-text	2	1.77 ± 0.5	1.71 ± 0.59	0.69
A 13		Aneg	1	0.83 ± 0.38	0.94 ± 0.25	0.22
A 14		R + Free-text	9	5.2 ± 1.79	5.58 ± 1.26	0.34
A14a			*2*	1.1 ± 0.55	1.1 ± 0.4	0.98
A14b			*3*	1.7 ± 0.84	1.71 ± 0.74	0.96
A14c			*2*	1.2 ± 0.71	1.23 ± 0.67	0.88
A14d			*2*	1.2 ± 0.71	1.55 ± 0.57	0.04
**Score Pathologies Salivary glands**			16	10.77 ± 1.74	10.97± 2.71	0.223
A 15		Kprim	4	3.63 ± 0.62	3.52 ± 0.93	0.56
A 16		R + Free-text	8	5.3 ± 1.42	5.55 ± 1.75	0.6
A16a			*2*	0.83 ± 0.65	0.9 ± 0.75	0.7
A16b			*2*	1.3 ± 0.54	1.39 ± 0.62	0.56
A116c			*2*	1.53 ± 0.63	1.48 ± 0.68	0.77
A116d			*2*	1.67 ± 0.61	1.77 ± 0.5	0.45
A 17		Kprim	4	1.8 ± 0.85	1.9 ± 0.75	0.62
