# Supplementary material for: Ultrasound education in the digital era: face-to-face vs. webinar-teaching of head and neck ultrasound theory—a prospective multi-center study
Source: Front Med (Lausanne). 2025 May 9;12:1506260. doi: 10.3389/fmed.2025.1506260 (PMC12098340; doi:10.3389/fmed.2025.1506260)
Supplement: Supplementary file 6 [file Data_Sheet_6.pdf]

**Supplement 6 Baselinecharacteristic and Results of the Theory-Test$^{post}$ of the validation group (beginner) and control + study group**

| | **Validation group (beginner)** | **Control + Study-group** | **p-value** |
|---|---|---|---|
| **Baselinecharacteristic** | | | |
| **Gender** | | | 0.004 |
| female (n;%) | 36 (77%) | 29 (48%) | |
| male (n;%) | 11 (23%) | 27 (44%) | |
| n.a. (n;%) | 0 | 5 (8%) | |
| **Age** ( mean±SD ) in years | 25.5 ± 3.3 | 32.7 ± 5.1 | < 0.001 |
| Ultrasound courses attended (regardless of type) | | | 0.14 |
| Yes (n;%) | 36 (77%) | 50 (82%) | |
| No (n;%) | 11 (23%) | 6 (10%) | |
| n.a. (n;%) | 0 | 5 (8%) | |
| **Number of ultrasound examinations** (regardless of area) (mean±SD ) | 0.7 ± 1.4 | 248 ± 317 | < 0.001 |
| **Theory-Test$^{post}$** | | | |
| **Total Score Theory-Test$^{post}$** (max. 54 points) | 20.8 ± 5.3 | 37.4 ±5.3 | < 0.001 |
| **Score Lymphnode** (max. 12 points) | 5.6 ± 1.8 | 9.5 ±1.8 | < 0.001 |
| **Score Cervical soft tissue** (max. 26 points) | 8.3 ± 3.5 | 17.2 ±3.0 | < 0.001 |
| Score salivary glands (max. 16 points) | 7.3 ± 2.4 | 10.9 ±2.3 | < 0.001 |
